# Mutational Studies of the Mersacidin Leader Reveal the Function of Its Unique Two-Step Leader Processing Mechanism

**DOI:** 10.1021/acssynbio.2c00088

**Published:** 2022-05-03

**Authors:** Jakob H. Viel, Oscar P. Kuipers

**Affiliations:** Department of Molecular Genetics, University of Groningen, Nijenborgh 7, 9747 AG Groningen, The Netherlands

**Keywords:** mersacidin, RiPP, lanthipeptide, leader, heterologous expression, E. coli, mutation

## Abstract

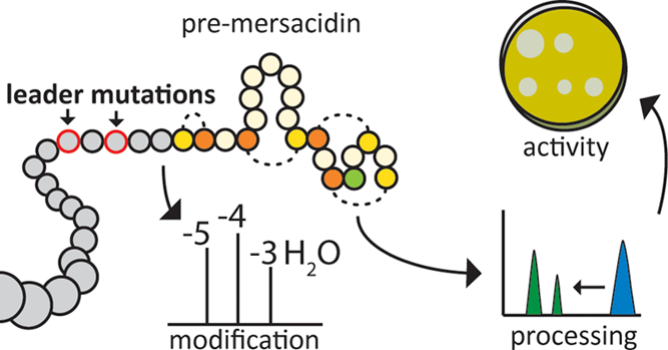

The class II lanthipeptide mersacidin, a ribosomally synthesized and post-translationally modified peptide (RiPP), displays unique intramolecular structures, including a very small lanthionine ring. When applied in the growing field of RiPP engineering, these can add unique features to new-to-nature compounds with novel properties. Recently, a heterologous expression system for mersacidin in *Escherichia coli* was developed to add its modification enzymes to the RiPP engineering toolbox and further explore mersacidin biosynthesis and leader-processing. The dedicated mersacidin transporter and leader protease MrsT was shown to cleave the leader peptide only partially upon export, transporting GDMEAA-mersacidin out of the cell. The extracellular *Bacillus amyloliquefaciens* protease AprE was shown to release active mersacidin in a second leader-processing step after transport. The conserved LanT cleavage site in the mersacidin leader is present in many other class II lanthipeptides. In contrast to mersacidin, the leader of these peptides is fully processed in one step. This difference with mersacidin leader-processing raises fundamentally interesting questions about the specifics of mersacidin modification and processing, which is also crucial for its application in RiPP engineering. Here, mutational studies of the mersacidin leader–core interface were performed to answer these questions. Results showed the GDMEAA sequence is crucial for both mersacidin modification and leader processing, revealing a unique leader layout in which a LanM recognition site is positioned downstream of the conserved leader-protease LanT cleavage site. Moreover, by identifying residues and regions that are crucial for mersacidin-type modifications, the wider application of mersacidin modifications in RiPP engineering has been enabled.

## Introduction

Mersacidin is a class II lanthipeptide, a ribosomally synthesized and post-translationally modified peptide (RiPP), produced by *Bacillus amyloliquefaciens.*(1−3) Mersacidin originally stood out because of its high activity against methicillin-resistant *Staphylococcus aureus* strains.^1,4,5^ And, while its antimicrobial properties have not become less relevant, recent advancements in lanthipeptide engineering^6−10^ also give the understanding of mersacidin biosynthesis relevance from an engineering perspective.

**Figure 1:**
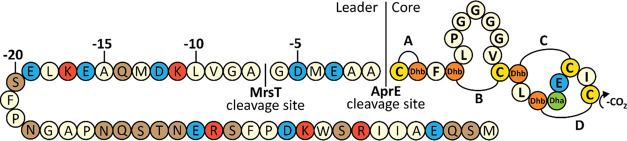
Mersacidin with leader peptide. Mersacidin’s C-terminal cysteine is decarboxylated by MrsD,^11,12^ after which MrsM dehydrates the threonine and serine residues of the core peptide and installs the lanthionine rings.^13,14^ MrsT exports the peptide and partially cleaves the leader peptide. The remaining part of the leader peptide is cleaved in the supernatant by the extracellular protease AprE.^23^

In mersacidin biosynthesis, the precursor MrsA is modified by MrsD, which decarboxylates the C-terminal cysteine,^11,12^ and MrsM, which dehydrates the serine and threonine residues of the core peptide and installs mersacidin’s four lanthionine rings.^13,14^ When the peptide is fully modified, MrsT cleaves part of the leader peptide and transports the core peptide, attached to the remaining part of the leader, out of the cell (Figure 1).^13,15,16^

The first ring of mersacidin, ring A, is particularly interesting. This uniquely small ring is formed in the opposite direction of mersacidin’s other three lanthionine rings and spans no additional amino acids. Due to the low profile of this smallest ring, it could be of particular interest to lanthipeptide engineering. The incorporation of lanthionine rings into foreign substrates using lanthipeptide modification machinery has been shown to increase peptide stability and resistance to proteolytic degradation while retaining their functionality.^17^ Introduction of mersacidin’s ring A into foreign substrates would provide them with these advantages while impacting their structure and function to the smallest amount possible for a lanthionine ring.

The application of mersacidin modifications in such an engineering purpose, requires a level of understanding of its modification machinery. The complete biosynthesis of mersacidin has been extensively characterized in its native producer *B. amyloliquefaciens.*(13,18−21) However, due to dependency on the transporter MrsT in these systems, a lack of product in the supernatant cannot be directly linked to a lack of modification by MrsM. Additionally, *B. amyloliquefaciens*’s many extracellular proteases^22^ obscure the exact mode of leader processing, and the production effort, as partially modified or unstable products would be readily degraded in the supernatant. For this purpose, another system was needed for more straightforward characterization and application of the mersacidin modification machinery.

A heterologous expression system in *Escherichia coli* was developed to produce a fully modified mersacidin precursor peptide (His6-MrsA).^15^ This system has already been applied to confirm that MrsT does not cleave the whole mersacidin leader, but cleaves before position −6 as predicted based on homology.^13,15^ The transported GDMEAA-mersacidin is inactive until the *B. amyloliquefaciens* extracellular protease AprE cleaves off the final six amino acids from the leader, releasing the fully modified and active peptide.^23^

**Figure 2:**
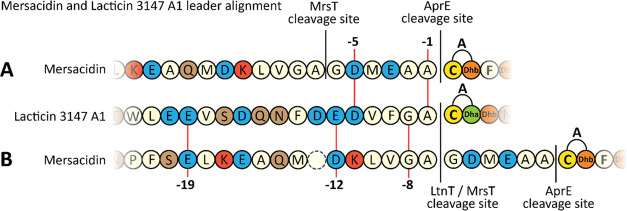
Mersacidin and lacticin 3147 A1 leader alignment. (A) The mersacidin leader aligned with the lacticin 3147 A1 leader. While the MrsT recognition site is out of sync with the LtnT recognition site, the alanine at position −1 and more noticeably the aspartate at position −5 are still conserved. (B) The mersacidin and lacticin 3147 A1 leaders aligned to their lanT cleavage sites. Well-conserved residues from the lanT recognition site like the negative charges at positions −19 and −12, as well as cleavage site GA are present in both leader peptides.

While lanthipeptide leader-processing by the host’s native extracellular proteases has been reported before,^24−26^ this mode of leader-processing is notable in the case of mersacidin. In reported cases of general proteases cleaving RiPP leaders, the biosynthetic gene cluster does not encode a dedicated leader protease,^24,26^ whereas the mersacidin leader is partially cleaved by MrsT upon transport.^13,15^ Moreover, in class II lanthipeptides that share the conserved LanT recognition- and cleavage site with mersacidin, the cleavage site is positioned at the end of the leader, resulting in fully processed peptide being transported out of the cell.^13^ This is especially striking in the case of Lacticin 3147 A1, which has a small first ring like mersacidin, but is fully processed by its LanT, LtnT^13,27−29^ (Figure 2).

The mersacidin leader thus appears to have a unique layout, where both a dedicated leader protease and a general protease are needed to produce active mersacidin.^23^ And, as the two-step leader processing has already been shown to have no function outside of the cell after transport,^23^ the question is raised what the functional importance of the GDMEAA sequence is in the interaction with mersacidin modification and leader-processing enzymes. Because this leader layout appears to be unique for mersacidin, addressing this question will be fundamentally of great interest. Additionally, it will be vital to the application of the mersacidin modification machinery in lanthipeptide engineering.

Hypothetically, the negative charges of the aspartate and glutamate residues perform a function in the interaction with one of the modification enzymes. Also, the six amino acids might be needed to accomplish a specific distance between parts of the mersacidin leader and the start of the core peptide, as has been reported to be crucial in the case of the FNLD box in class I lanthipeptides.^30−32^

To test these hypotheses, a range of mersacidin leader mutants was expressed in *E. coli* together with MrsMD. These mutants include a range of single and multiple specific residue mutants, including partial and complete deletion of GDMEAA, to assess the importance of specific residues in the GDMEAA sequence. Additionally, systemic single residue deletions and complementary alanine substitutions were made, confined to the leader–core interface, to investigate the importance of the distance between regions of the leader peptide, the GDMEAA sequence, and the start of the core peptide. The modification efficiency, and AprE cleaving efficiency, for all mutants was investigated by antimicrobial activity tests, and mass spectrometry analysis. Additionally, the ability of MrsT to recognize and cleave these leader mutants was assessed by proteolytic essays with the purified proteolytic domain of MrsT, MrsT150-His.^15^

## Results and Discussion

### Role of GDMEAA in Modification by MrsM

**Figure 3:**
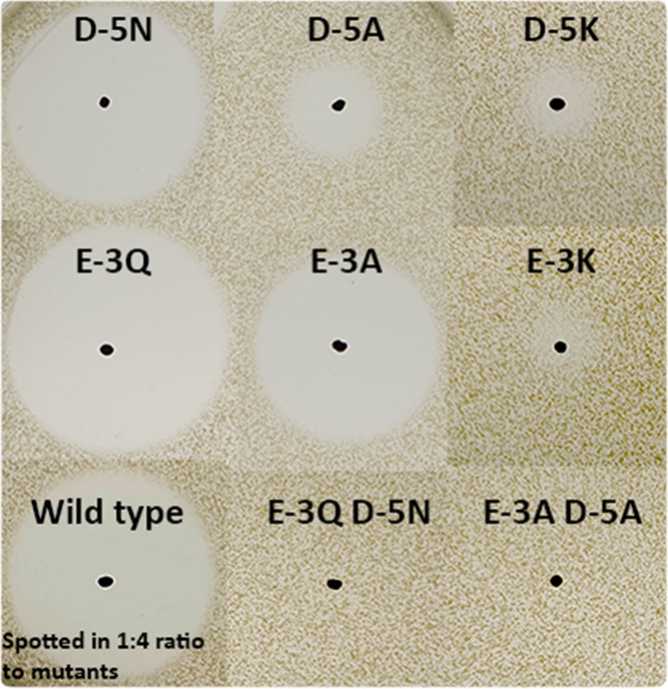
Activity comparison of different E-3 and D-5 mutants against *Micrococcus flavus.* E-3Q has 30–40% activity compared to the wild type, which is spotted in a 1:4 ratio here compared to the mutants (S4). Similar mutations of D-5 affect activity more than those of E-3. When E-3 or D-5 are substituted by the positively charged lysine residue, a very small amount of activity can still be detected. When D-5 and E-3 are substituted concurrently, no activity is detected.

**Figure 4:**
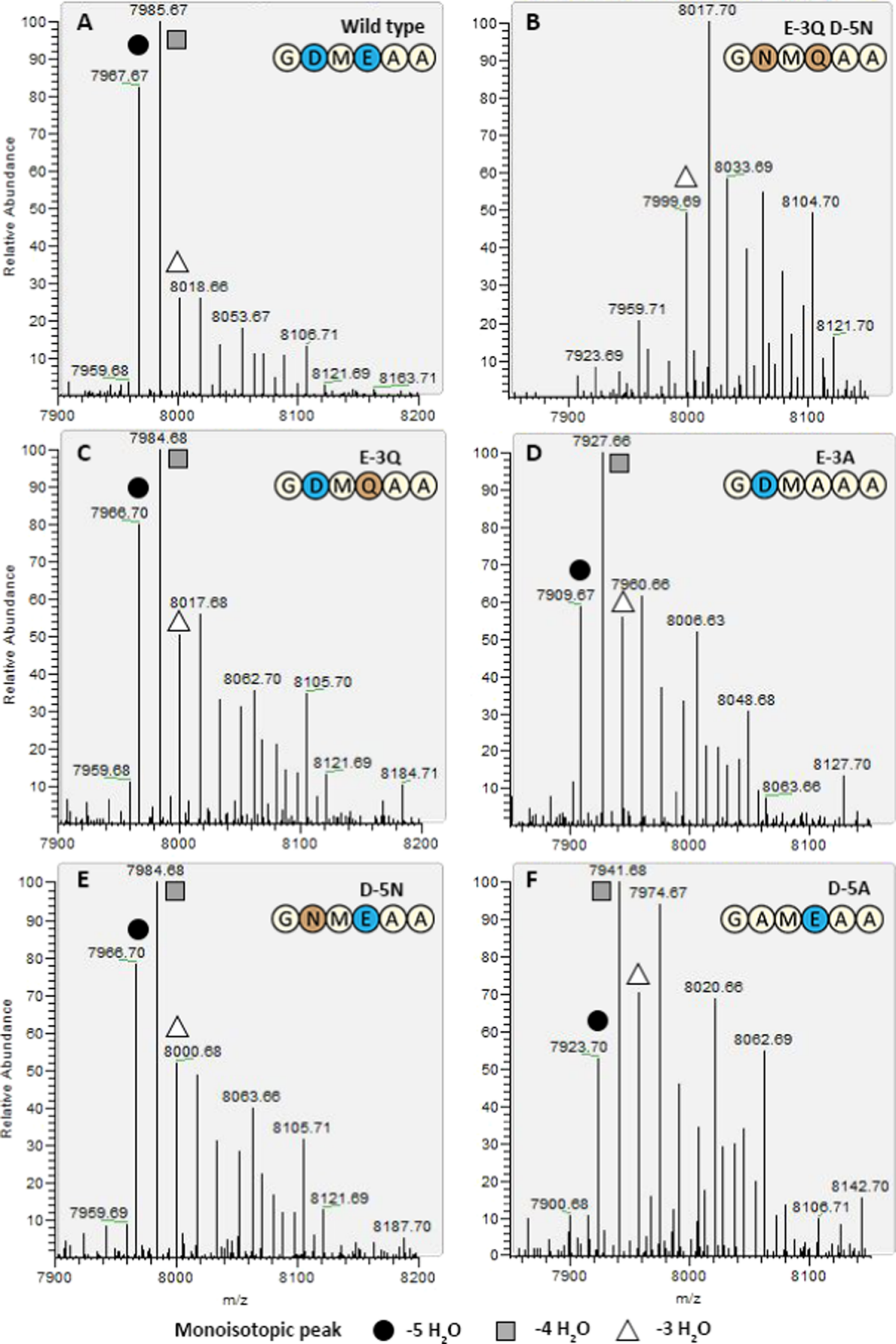
Ratios of different modification efficiencies of D-5 and E-3 mutants (LC-MS, monoisotopic). (A) The wild type has mostly 4× dehydrated and fully dehydrated His6-MrsA (theoretical 7967.65 Da). (B) When both D-5 and E-3 are substituted by their respective polar analogues, only three dehydrations are performed (theoretical 8001.70 Da). The loss of 2 Dalton suggests the free cysteines formed a disulfide bridge. The same is seen for E-3A D-5A (not shown). (C, D) E-3Q results in more 3× and 2× dehydrated peptide relative to the fully hydrated peptide, although the fully dehydrated peptide (theoretical 7966.66 Da) is still the second most abundant. For E-3A, this effect is seen even stronger, and 3× and 2× dehydrated peptides are each as abundant as fully dehydrated His6-MrsA (theoretical 7909.64 Da). (E, F) For D-5 mutants, the same effects are seen as for E-3 mutants. In the case of D-5A, there is a stronger decrease in dehydration efficiency than for E-3A (S3).

First, the importance of the negatively charged residues in the GDMEAA sequence was investigated by mutating residues D-5 and E-3 (Table 1, *b–i*). Mutation of residue E-3 and especially D-5 leads to a significant decrease in the production of fully modified His6-MrsA, when they were substituted for their respective polar analogues (E-3Q or D-5N) (Table 1 and Figure 3). This effect was seen even more strongly when either of these residues was substituted by an alanine (E-3A or D-5A), decreasing the production of fully modified His6-MrsA by 95% in the case of D-5A compared to the wild-type leader (Figure 3, S4). The liquid chromatography-mass spectrometry (LC-MS) data of these mutants supports the observed decrease in activity, showing a corresponding decrease in modification rate (Figure 4). When both negative residues are simultaneously substituted by their respective polar analogues, or by alanines, no antimicrobial activity is detected. LC-MS analysis of these mutants revealed that they are dehydrated at most three out of a possible five times, with trace amounts of peptide having four dehydrations (Figure 4, S3). While both negatively charged residues are important for mersacidin modification, mutation of D-5 resulted in lower detected antimicrobial activity than mutation of E-3. Since the dehydration ratios (Figure 4) and production yields (S2) are similar for the D-5 and E-3 mutants, the lower antimicrobial activity of D-5 mutants is likely due to lower ring forming efficiency. Notably, at least one of the negatively charged residues has to be present for full modification the occur.

**Table 1:**
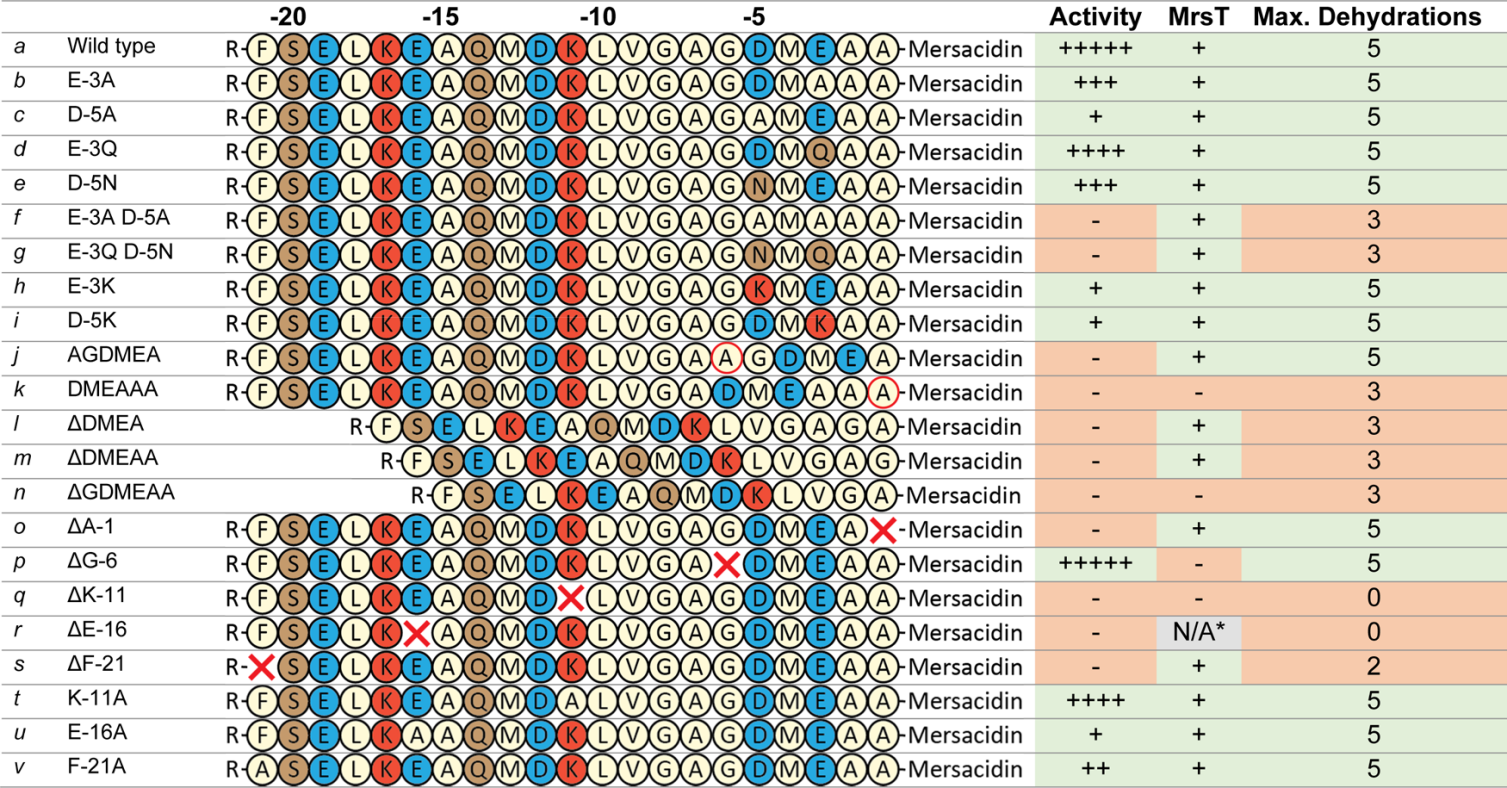
Mersacidin Leader Mutants Tested in This Study^a^

a Color coding of residues: brown: polar, blue: negatively charged, red: positively charged, cream: hydrophobic/glycine, red outline: residue addition, red cross: residue deletion. * For ΔE-16, the yield was not sufficient to assess MrsT150-His cleavage.

Respective mutations of negatively to positively charged residues, D-5K and E-3K, drastically lowered the modification rate as expected. Surprisingly, a small fraction of the substrate is still fully modified and active (Figure 3). Indicating that as long as one of the negative charges remains in place, some fully modified His6-MrsA can be produced (S4). Additionally, the detection of antimicrobial activity shows that *B. amyloliquefaciens* AprE is still able to process these mutant leaders, even though they include major changes close to its cleavage site.

Having established that the negative charges of the GDMEAA linker play a vital role in at least one of the mersacidin modification steps, the importance of the exact position of these negative charges relative to the core peptide was explored. Leader mutants were created in which D-5 and E-3 are shifted either one step to the N- or C-terminus (DMEAAA and AGDMEA) (Table 1, *j*,*k*).

**Figure 5:**
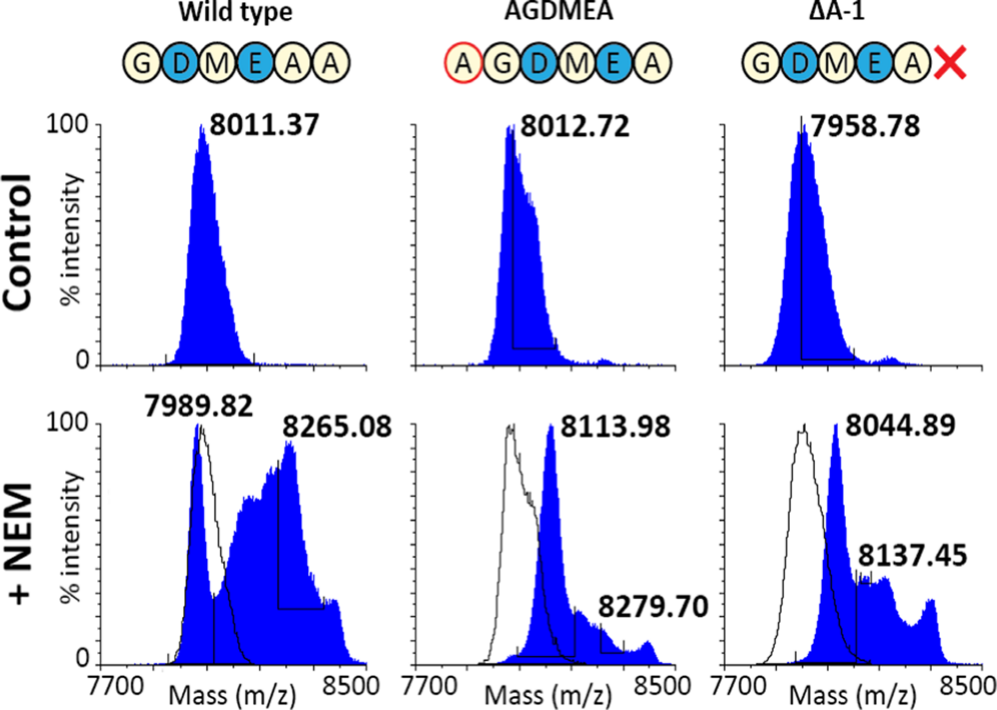
MALDI-TOF analysis of free cysteine assays on fully dehydrated but inactive mutants.An *N*-ethylmaleimide (NEM) free cysteine essay was done for the mutants AGDMEA and Δ-1, which could be fully dehydrated (S3) but showed no activity upon leader cleavage (S4). The differently dehydrated species appear as centroid peaks in MALDI-TOF analysis of molecules this size. A shift of the most dehydrated peak to the right means that not all rings are formed. Compared to the wild type, where part of the fully dehydrated species was unaffected by the free cysteine essay, the masses of all the peptide of both tested mutants experienced a shift approximating at least one free cysteine (125 Da shift). Both mutants were thus not fully modified, explaining why they showed no antimicrobial activity upon leader removal.

The AGDMEA mutant, in which the negative charges are moved to positions E-2 and D-4, was fully dehydrated and well produced (S2 and S3). However, no antimicrobial activity could be detected upon cleavage by AprE. Since this mutation is inside the AprE cleavage site, the lack of activity could have hypothetically been caused by an inability of the protease to fully cleave the leader peptide. However, digestion patterns of AprE-processed wild-type and mutant peptide were inspected by Matrix-assisted laser desorption/ionization-time of flight (MALDI-TOF) analysis (S5), and a lack of processing by AprE at the leader–core interface was not detected. Finally, an *N*-ethylmaleimide (NEM) free cysteine essay revealed that only three out of four rings were formed, explaining the lack of antimicrobial activity (Figure 5)(S4).

**Figure 6:**
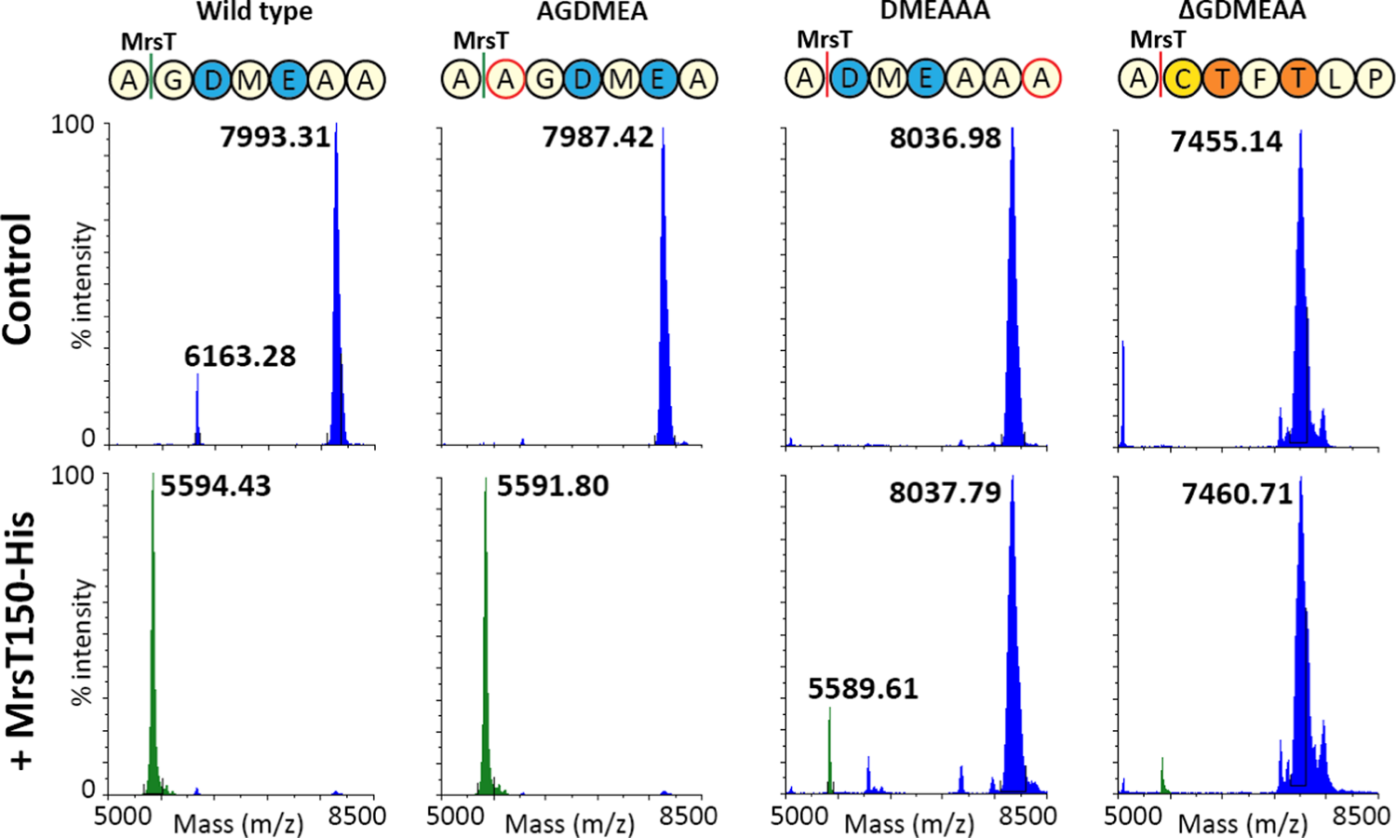
MALDI-TOF analysis of MrsT150-His digested leader mutants. Mutants with a glycine or an alanine residue after the MrsT cleavage site are digested by MrsT150-His, resulting in the detection of the mersacidin leader mass without GDMEAA (green, 5590.10 Da theoretical average). Variants with an aspartate or cysteine residue after the MrsT cleavage site remain largely undigested. Masses of the uncleaved peptides were acurately measured by LC-MS (S3), which in these MALDI-TOF analysis spectra appear as centroid masses of differently dehydrated species.

**Figure 7:**
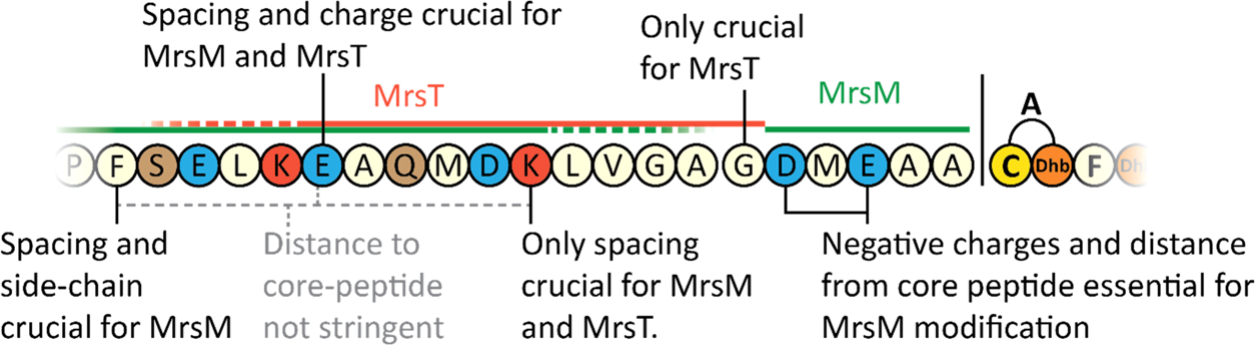
Overview of specific leader residue function. The mersacidin leader has a unique layout, where a LanM recognition site is positioned downstream of the LanT leader cleavage site.

Increasing the distance between the negative charges and the core peptide (DMEAAA) led to only three out of five dehydrations occurring. Additionally, in this construct the aspartate is in position 1 of the MrsT cleavage site (Figure 6), leading to a drastic decrease in MrsT150-His cleaving efficiency, which is discussed later. It is noteworthy that although residue D-5 is more important for modification efficiency than E-3, shifting the negative charges to positions −6 and −4 has a more detrimental effect on modification efficiency than shifting them to positions −2 and −4 (Figure 7).

Next, mutants in which the GDMEAA sequence was fully or largely deleted were studied (Table 1, *j*,*k*). Interestingly, in these mutants, three out of five dehydrations could still occur (S3), as was observed when both negative residues were substituted simultaneously, or shifted toward the N-terminus. These results strongly point toward the GDMEAA sequence playing a role in a specific enzyme–substrate interaction that is required for a particular dehydration or cyclization step, rather than playing a role in MrsM-substrate binding.

Correspondingly, in the core peptide, residue E17 is at position −3 relative to C20. In previous reports, there has been some speculation on the role of this negative charge in mersacidin, like on its role in the mechanism of antimicrobial activity.^19,33^ The new insights made here allow for the hypothesis on an additional role of E17. Namely, the involvement of its negative charge in the more efficient formation of ring D. Although mutational studies have shown E17 can be mutated to several other polar or charged residues, abolishing activity,^19^ substitutions with hydrophobic residues at most result in trace amounts of product. The decrease or absence of production when mutating this residue surely points toward a role of E17 in modification efficiency. And the importance of the negative charges in D-5 and E-3 demonstrated here suggests that E17 might also be involved in an interaction with MrsM during the maturation process.

Additionally, the negativly charged residues in the GDMEAA sequence and the core peptide might interact to prevent the formation of secondary structures in the core peptide that interfere with efficient modification, e.g., disulfide bridges. Structural analysis of mersacidin shows that the N- and C-termini are positioned near each other in the fully matured peptide.^34,35^ And, while remaining speculative, the constructs lacking residues D-5 and E-3 contain a disulfide bridge according to their −2 Da mass difference (S3). The formation of a disulfide bridge is also in line with the three out of five dehydrations that are detected when both D-5 and E-3 are mutated, as two cysteine residues in the core peptide would not be part of a lanthionine ring.

To confirm that any lack of activity from cleaved peptides is not caused by a lack of AprE processing, MALDI-TOF MS analyses were performed for all processed peptides (S5). The data showed that leader processing at the leader–core interface was not visibly impaired, confirming the broad substrate specificity of AprE.^36^ Finally, it should be noted that although modification efficiency is known to strongly affect the total production yield, changes in codon usage can have affected production to some extent. However, since different approaches in, e.g., removing the negative charges D-5 and E-3 lead to very similar yields in D-5A E-3A, D-5N E-3Q, ΔDMEA, ΔGDMEAA, and ΔGDMEAA, codon usage does not seem to have a measurable effect on production.

### Role of GDMEAA in MrsT Cleavage

To assess the ability of MrsT to recognize and process the mutated leader variants, they were incubated with MrsT150-His, the heterologously expressed proteolytic domain of MrsT.^15^ After incubation, the samples were analyzed by MALDI-TOF MS, which showed MrsT150-His was able to cleave most of the tested mutants. Also, it was able to cleave at its recognition site in the mersacidin leader when the core peptide had been deleted (S8). This shows that the leader-recognition site and proteolytic domain of MrsT do not require any interaction with a partially or fully modified mersacidin core structure to process the substrate. And so, cleavage of the separate leader peptide functions as a control to assure that the observed cleaving efficiency is not influenced by the modification rate of the mutants.

As mentioned above, the aspartate residue at position 1 of the MrsT cleavage site in the DMEAAA mutant leads to a drastic decrease in the MrsT150-His cleaving efficiency. To explore this observed effect on MrsT cleavage further, a ΔG-6 mutant was created, leaving the original distance from D-5 and E-3 to the core peptide intact. As expected, this mutant could not be cleaved by MrsT150-His, confirming MrsT cannot cleave before an aspartate residue. Surprisingly, the modification efficiency of this mutant was as high as in the wild type, and it showed the same level of antimicrobial activity. This suggests that the only function of the G-6 residue is as a spacer for MrsT processing, and it is likely crucial for the mersacidin transportation process. Possibly, the GAG sequence at the MrsT cleavage site is also required to give this region flexibility for efficient recognition and processing by MrsT.

Additionally, MrsT150-His was not able to cleave when the full GDMEAA sequence was deleted, meaning MrsT cannot cleave with a cysteine in position 1 of its cleavage site. This is a notable observation. The leader of Lacticin 3147 A1, the only known RiPP that has a CS-ring quite similar to mersacidins CT-ring, can be completely cleaved by its protease and transporter LtnT (Figure 2).^13,29^

Interestingly, a recent genome analysis of the *Bacteroidetes* phylum predicts a range of class I lanthipeptides to be processed in a two-step mechanism resembling that of mersacidin.^37^ These lanthipeptide gene clusters encode a class II lanthipeptide-like transporter composed of a proteolytic and transmembrane domain. This suggests that two-step leader processing, as seen in mersacidin, may be underreported, although the mechanistic necessity of this kind of processing could well differ between different lanthipeptide biosynthesis systems employing this mechanism.

### Role of Specific Residues of the Leader and their Distance the Core Peptide

To investigate the role of specific residues of the leader and their distance from the core peptide, systematic single amino acid deletion mutants (Table1, *o*–*s*) were tested along with complementary alanine substitution mutants (Table 1, *t*–*v*). The deletions all resulted in a complete loss of antimicrobial activity with the exception of ΔG-6. Deletion of A-1 leads to full dehydration but incomplete ring formation, as was observed for AGDMEA (Figure 5). The deletion of K-11 and E-16 resulted in no dehydrations occurring at all, indicating that this region is crucial for MrsM recognition. In contrast to the deletions, for all of the alanine substitutions at least a small amount of substrate was still fully modified. However, antimicrobial activity in E-16A and F-21A (S4) was strongly reduced compared to the wild type. The efficient modification of ΔG-6 shows that the distance from residues, upstream of D-5, to the core peptide is not responsible for the lack of modification in the other single amino acid deletions ΔF-21, ΔE-16, and ΔK-11. However, the spacing appears to play an important role in leader secondary structure formation, e.g., alfa helix formation. Substitution of the deleted residues by alanine residues keeps the spacing similar but appears to lower the leader specificity, leading to a lower modification efficiency. Strikingly, the only tested residue that could be deleted from the leader sequence without affecting MrsM modification efficiency is ΔG-6, which is positioned in the GDMEAA region that is shown here to be essential for both modification and leader processing.

The elucidation of mersacidin’s peculiar modification and leader-processing parameters points out potential effects occurring in the heterologous expression of lanthipeptides of unknown function, like those acquired from genome mining. Additional processing after export might occur under natural conditions, leading to inactive peptides upon heterologous expression.^23^ Additionally, it is common practice to substitute the leader cleavage site with that of a well-established lanthipeptide, like the cleavage site of NisP,^38^ LahT150,^16^ or that of a commercially available protease.^39^ While this approach is versatile and convenient, it might replace residues in the original leader that are essential for a specific modification, like the substitution of D-5 and E-3 in the mersacidin leader prevents a specific modification from occurring while the rest of the peptide is still modified. These effects could obscure any activity the mature peptide might have.

Finally, it would be interesting to determine exactly what modification or modifications are dependent on the GDMEAA linker. An obvious candidate would be ring A, with its unusual size, direction of formation, and closest position to GDMEAA. Lacticin 3147 A1 with its similar first ring structure does not require a GDMEAA-like sequence to form its first ring, although the negatively charged residue at position −5 is conserved in the leader of both peptides (Figure 2). Additionally, because of the globular structure of mersacidin, it cannot be ruled out that GDMEAA plays a role in the formation of rings C and D. Further investigation into this topic, to find out what parts of the leader could be omitted or mutated in case not all rings are required, would also be beneficial for the purpose of RiPP engineering. Ultimately, production of the small ring A by itself, with and without the D-5 and E-3 mutations described here, would prove the applicability of the mersacidin system in RiPP engineering and elucidate the prerequisites for its formation.

## Conclusion

The GDMEAA sequence is crucial for the full modification of mersacidin by MrsM and for leader processing by MrsT. Specifically, the negatively charged residues E-3 and D-5 are crucial for two dehydrations and at least one ring formation to occur. Residue G-6 is crucial for leader cleavage by MrsT and possibly for flexibility during the transport process. The unique leader layout revealed here is not only fundamentally interesting, but also gives ample direction for future application of mersacidin modification enzymes in RiPP engineering.

## Materials and Methods

### Bacterial Strains and Growth Conditions

*E. coli* was used for all cloning (TOP10) and expression (BL21(DE3)) purposes. It was grown in LB broth (Foremedium) at 37 °C shaking at 225 rpm, or on LB agar (Foremedium) at 37 °C unless stated otherwise. Growth media for *E. coli* strains containing the plasmids pACYC or pBAD was supplemented with 15 μg/mL chloramphenicol or 100 μg/mL, respectively. *M. flavus* was used as the indicator strain in all antimicrobial activity tests. It was grown in LB broth at 37 °C shaking at 225 rpm, unless stated otherwise.

### Molecular Cloning

All molecular cloning was done using previously described methods,^40^ using protocols provided by the manufacturer unless stated otherwise. The plasmids used in this study were constructed from those previously described,^15^ using mutagenic primers (Biolegio, Nijmegen, The Netherlands) (S9). In all cases, mutations were introduced using round PCR on the template using Phusion polymerase (Thermo Scientific), in which the mutations were introduced, as well as complementary Eco31I restriction sites. The cleaned up (NucleoSpin Gel and PCR Clean-up, Macherey-Nagel) PCR product was digested with FastDigest Eco31I (Thermo Scientific). After an additional clean-up step, the digested DNA was scarlessly ligated with T4 ligase (Thermo Scientific) using the complementarity Eco31I overhangs.

*E. coli* TOP10 was subsequently transformed with the ligation mixture. A selection of the resulting colonies was picked up in liquid medium and grown overnight. The resulting cultures were used to isolate the plasmids (NucleoSpin Plasmid EasyPure, Macherey-Nagel), which were sent for sequencing (Macrogen Europe, Amsterdam, The Netherlands) and to prepare glycerol stocks.

### Peptide and Protein Production

For all production purposes, *E. coli* BL21(DE3) was freshly transformed with the required plasmids. In the case of the mersacidin leader mutants, these were pACYC containing the mutant His6-MrsA and MrsM, and pBAD MrsD.^15^ For the production of MrsT150-His, pACYC MrsT150-His^15^ was used. For the expression of His6-MrsA and each of its mutants, several freshly transformed colonies were picked up and grown overnight. The overnight cultures were diluted 1:100, in 300 mL of fresh medium, and grown for 145 min. The expression cultures were then cooled in an ice bath to 16 °C and induced with 1 mM IPTG (pACYC) and/or 0.2% arabinose (pBAD). Finally, the cultures were incubated at 16 °C, shaking at 225 rpm, for 29 h before being harvested. Expression of MrsT150-His and AprE-His was obtained as described previously.^15,23^

### Peptide and Protein Purification

After harvesting the expression cultures, the pellets were washed once in 25 mL of binding buffer (20 mM H_2_NaPO_4_ (Merck), 0.5 M NaCl (VWR), and 20 mM imidazole (Merck), pH 7.4) and then resuspended in 10 mL of binding buffer. The resuspended cells were lysed by sonication and spun down. The peptide or protein was then purified from the supernatant by Ni-NTA chromatography, using 0.9 mL of Ni-NTA agarose slurry (Qiagen) (CV = ca. 0.5 mL). After loading the sample, the column was washed with 10 column volumes (CV) of binding buffer, followed by washing by a 5 CV wash buffer (20 mM H_2_NaPO_4_, 0.5 M NaCl, 50 mM imidazole, pH 7.4). Finally, the sample was eluted with 1.8 mL of elution buffer (20 mM H_2_NaPO_4_, 0.5 M NaCl, 500 mM imidazole, pH 7.4). When purifying MrsT150-His and AprE-His, the elution buffer contained 250 mM imidazole.

His6-MrsA and its leader mutants were further purified by reversed-phase chromatography using an open C-18 column with 0.25 g (CV = 1 mL) of 55–105 μm C18 resin (Waters). The column was wetted with 2 CV acetonitrile (ACN)(VWR) + 0.1% trifluoroacetic acid (TFA)(Sigma) and then equilibrated with 5 CV Milli-Q + 0.1% TFA. The samples eluted from Ni-NTA chromatography were acidified with 0.5% TFA until pH < 4 and loaded onto the column. After a 10 CV wash (Milli-Q + 0.1% TFA), followed by a 5 CV wash (20% ACN + 0.1% TFA), the sample was eluted in 4 CV 50% ACN + 0.1% TFA. The elution fraction was freeze-dried, resulting in a semipure peptide, which was stored at −20 °C until further use.

### Tricine-SDS-Page

Tricine-SDS-page gels were prepared and run as previously described.^41^ The freeze-dried C-18 elution fractions were dissolved in 150 μL of Milli-Q water; 4 μL of this solution was mixed with 3.5 μL of loading dye (550 mM dithiothreitol (Sigma-Aldrich), 250 mM Tris-HCl (Boom), 50% glycerol (Boom), 10% sodium dodecyl sulfate (Sigma-Aldrich), 0.5% Coomassie Blue R-250 (Bio-Rad), pH 7.0), and 6.5 μL of Ni-NTA chromatography elution buffer. The samples were boiled for 5 min, after which they were run alongside a prestained ladder (PageRuler, Thermo Scientific).

### Digestion with MrsT150-His and AprE-His

Both His-tagged proteases were used for digestion in their Ni-NTA chromatography elution buffer, containing 250 mM imidazole. All digests were done in a final volume of 10 μL, of which 1 μL of the respective protease, and an amount of peptide depending on its concentration, supplemented with Milli-Q water up to 10 μL. The digests were incubated at 37 °C for 1 h in the case of AprE-His, and 3 h in the case of MrsT150-His.

### NEM Free Cysteine Essay

*N*-Ethylmaleimide (NEM) free cysteine assays were performed to determine ring formation efficiency. As a control, semipure fully modified His6-MrsA was used, which has been shown previously to be approximately 20–35% fully modified. The tested peptides were added in equal amounts approximated by the yield, as determined by tricine-SDS-page. The control was added in a 2:1 ratio. The volume of all tested samples was first set to 8 μL by adding Milli-Q water. The samples were then diluted 1:1 by adding 8 μL of 200 mM phosphate-buffered saline (Boom). To each sample, 5 mg/mL Tris(carboxyethyl)phosphine hydrochloride (TCEP)(Sigma) was added, after which they were incubated at room temperature for 20 min. Then, 4.5 μL of 25 mg/mL NEM (Sigma) was added followed by a 1.5 h incubation. Finally, the samples were purified by ZipTip (Merck), using the manufacturer’s protocol, and eluted in 30 and 60% ACN + 0.1% TFA. The elution fractions were analyzed by MALDI-TOF MS.

### Mass Spectrometry

MALDI-TOF MS analysis of all unprocessed and processed peptides was done as described previously.^42^ The unprocessed more concentrated peptides were spotted in several dilutions, approximately 5 and 25 times diluted in Milli-Q water. The dilution giving the highest signal was used.

LC-MS of the semipure unprocessed His6-MrsA and mutants was done on their open C-18 elution fractions, which were dissolved in 150 μL of Milli-Q water after freeze-drying, as described previously.^15^ Both the average and monoisotopic masses were extracted for analysis (S3 and S7).

### Antimicrobial Activity Tests

For all antimicrobial activity tests, the peptide was digested by AprE-His as described above. Of the digests, 9 μL was spotted on an antimicrobial activity plate, leaving 1 μL for MALDI-TOF analysis. Antimicrobial activity plates were prepared by diluting an overnight culture of *M. flavus* 1:100 in a hand warm 50/50 mixture of LB broth and LB agar and then adding 12 mL of this mixture to 90 mm Petri dishes.

Because of the large range of different activities for the respective leader mutants, antimicrobial activity tests were done by a stepwise decrease of peptide used in multiple rounds, to increase the resolution and the robustness of the results. Fully modified semipure His6-MrsA from three separate expressions was mixed in equal amounts to form an averaged control. Of this control sample, 2 μL was digested and spotted on every plate to normalize halo sizes and to verify AprE-His activity. For His6-MrsA, it was found that spotting 2 μL creates a halo of medium size, which allows for the best accuracy in quantification (S4). Because some of the mutants show no or very low activity, all of the leader mutants were spotted in a 4:1 ratio to the control by digesting 8 μL of each peptide and spotting them on a plate with 2 μL of positive control. Samples that showed high enough activity to produce a halo as large as the 2 μL control were tested again, this time using 4 μL, leading to a 2:1 ratio. Finally, the most active samples were tested in equal amounts (2 μL) and directly compared to the positive control (S4).

## Supporting Information Available

Full amino acid sequence of all mutants (S1); yield determination by Tricine-Page (S2); LC-MS analysis of all mutants (S3); antimicrobial activity tests of all mutants (S4); spectra of AprE cleaved mutants (S5); comparison of AprE digestion patterns wild type, AGDMEA, and ΔA-1 (S6); table of theoretical average masses (S7); MrsT150-His cleavage analysis (S8); and list of primers used in this study (S9) (PDF)
