# Supplementary material for: Mutational Studies of the Mersacidin Leader Reveal the Function of Its Unique Two-Step Leader Processing Mechanism
Source: ACS Synth Biol. 2022 May 3;11(5):1949–57. doi: 10.1021/acssynbio.2c00088 (PMC9127955; doi:10.1021/acssynbio.2c00088)
Supplement: Supplementary file 1 — sb2c00088_si_001.pdf [file sb2c00088_si_001.pdf]

Supporting information to:

Mutational studies of the mersacidin leader reveal the function of its unique two-step leader processing mechanism.

Jakob H. Viel, Oscar P. Kuipers*

Department of Molecular Genetics, University of Groningen, Nijenborgh 7, 9747 AG, Groningen, The Netherlands.

***Correspondence:**
Oscar P. Kuipers
o.p.kuipers@rug.nl

Keywords: Mersacidin, RiPP, lanthipeptide, leader, heterologous expression, *E. coli,* mutation

Contents

SI 1. Full amino acid sequence of all mutants 3
SI 2. Yield determination by Tricine-Page 4
SI 3. LC-MS analysis of mutants 5
SI 4. Antimicrobial activity tests of all mutants 9
SI 5. Spectra of AprE cleaved mutants 12
SI 6. Comparison of AprE digestion patterns wild type, AGDMEA and ΔA-1 23
SI 7. Table of theoretical average masses 24
SI 8. MrsT150-His cleavage analysis 25
SI 8. List of primers used in this study 33
References 34

S1. Full amino acid sequence of all mutants

		**Sequence**
a	**Wild type (His6-MrsA)**	MHHHHHHGSQEAIIRSWKDPFSRENSTQNPAGNPFSELKEAQMDKLVGAGDMEAACTFTL<br>PGGGGVCTLTSECIC
b	pACYC E-3A + MrsM	MHHHHHHGSQEAIIRSWKDPFSRENSTQNPAGNPFSELKEAQMDKLVGAGDMAAACTFTL<br>PGGGGVCTLTSECIC
c	pACYC D-5A + MrsM	MHHHHHHGSQEAIIRSWKDPFSRENSTQNPAGNPFSELKEAQMDKLVGAGAMEAACTFTL<br>PGGGGVCTLTSECIC
d	pACYC E-3Q + MrsM	MHHHHHHGSQEAIIRSWKDPFSRENSTQNPAGNPFSELKEAQMDKLVGAGDMQAACTFTL<br>PGGGGVCTLTSECIC
e	pACYC D-5N + MrsM	MHHHHHHGSQEAIIRSWKDPFSRENSTQNPAGNPFSELKEAQMDKLVGAGNMEAACTFTL<br>PGGGGVCTLTSECIC
f	pACYC E-3A D-5A + MrsM	MHHHHHHGSQEAIIRSWKDPFSRENSTQNPAGNPFSELKEAQMDKLVGAGAMAAACTFTL<br>PGGGGVCTLTSECIC
g	pACYC E-3Q N-5A + MrsM	MHHHHHHGSQEAIIRSWKDPFSRENSTQNPAGNPFSELKEAQMDKLVGAGNMQAACTFTL<br>PGGGGVCTLTSECIC
h	pACYC E-3K + MrsM	MHHHHHHGSQEAIIRSWKDPFSRENSTQNPAGNPFSELKEAQMDKLVGAGDMKAACTFTL<br>PGGGGVCTLTSECIC
i	pACYC D-5K + MrsM	MHHHHHHGSQEAIIRSWKDPFSRENSTQNPAGNPFSELKEAQMDKLVGAGKMEAACTFTL<br>PGGGGVCTLTSECIC
j	pACYC AGDMEA + MrsM	MHHHHHHGSQEAIIRSWKDPFSRENSTQNPAGNPFSELKEAQMDKLVGAAGDMEACTFTL<br>PGGGGVCTLTSECIC
k	pACYC DMEAAA + MrsM	MHHHHHHGSQEAIIRSWKDPFSRENSTQNPAGNPFSELKEAQMDKLVGADMEAAACTFTL<br>PGGGGVCTLTSECIC
l	pACYC ΔDMEA + MrsM	MHHHHHHGSQEAIIRSWKDPFSRENSTQNPAGNPFSELKEAQMDKLVGAGACTFTL<br>PGGGGVCTLTSECIC
m	pACYC ΔDMEAA + MrsM	MHHHHHHGSQEAIIRSWKDPFSRENSTQNPAGNPFSELKEAQMDKLVGAGCTFTL<br>PGGGGVCTLTSECIC
n	pACYC ΔGDMEAA + MrsM	MHHHHHHGSQEAIIRSWKDPFSRENSTQNPAGNPFSELKEAQMDKLVGACTFTL<br>PGGGGVCTLTSECIC
o	pACYC ΔA-1 + MrsM	MHHHHHHGSQEAIIRSWKDPFSRENSTQNPAGNPFSELKEAQMDKLVGAGDMEACTFTL<br>PGGGGVCTLTSECIC
p	pACYC ΔG-6 + MrsM	MHHHHHHGSQEAIIRSWKDPFSRENSTQNPAGNPFSELKEAQMDKLVGADMEAACTFTL<br>PGGGGVCTLTSECIC
q	pACYC ΔK-11 + MrsM	MHHHHHHGSQEAIIRSWKDPFSRENSTQNPAGNPFSELKEAQMDLVGAGDMEAACTFTL<br>PGGGGVCTLTSECIC
r	pACYC ΔE-16 + MrsM	MHHHHHHGSQEAIIRSWKDPFSRENSTQNPAGNPFSELKAQMDKLVGAGDMEAACTFTL<br>PGGGGVCTLTSECIC
s	pACYC ΔF-21 + MrsM	MHHHHHHGSQEAIIRSWKDPFSRENSTQNPAGNPSELKEAQMDKLVGAGDMEAACTFTL<br>PGGGGVCTLTSECIC
t	pACYC K-11A + MrsM	MHHHHHHGSQEAIIRSWKDPFSRENSTQNPAGNPFSELKEAQMDALVGAGDMEAACTFTL<br>PGGGGVCTLTSECIC
u	pACYC E-16A + MrsM	MHHHHHHGSQEAIIRSWKDPFSRENSTQNPAGNPFSELKAAQMDKLVGAGDMEAACTFTL<br>PGGGGVCTLTSECIC
v	pACYC F-21A + MrsM	MHHHHHHGSQEAIIRSWKDPFSRENSTQNPAGNPASELKEAQMDKLVGAGDMEAACTFTL<br>PGGGGVCTLTSECIC

S2. Yield determination by Tricine-Page

The freeze-dried C-18 elution fraction of each peptide was dissolved in 150 µL of Milli-Q water, 4 µL of this solution was run on gel. Thus, effectively 8 mL of expression culture was run per well in case of 100 % purification efficiency. Non annotated wells contain peptides not discussed in this study.

Gel 1

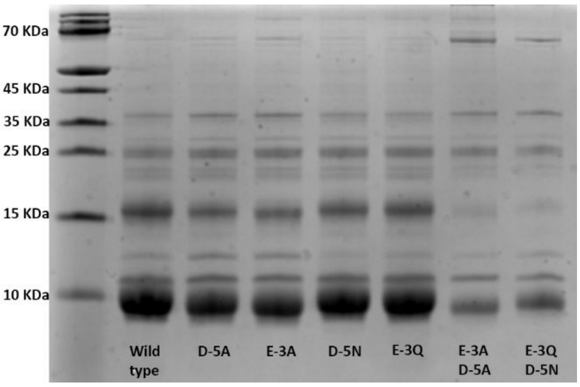

Gel 2

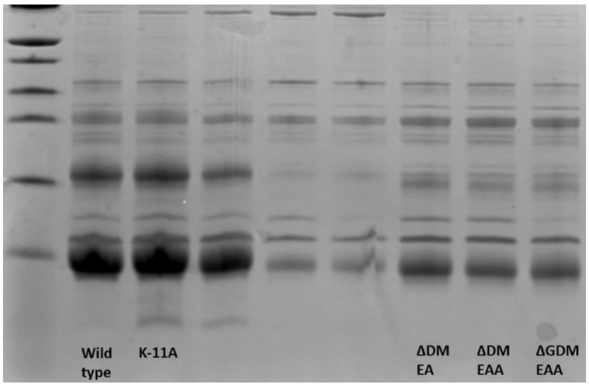

Gel 3

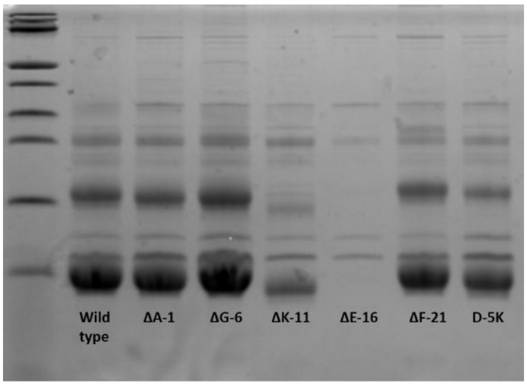

Gel 4

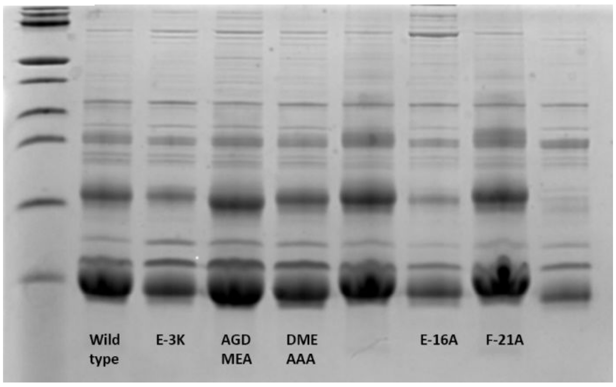

S3. LC-MS analysis of mutants

All masses are the MrsD decarboxylated masses

<table>
<tr><th></th><th>Mutant</th><th>Spectrum</th><th>$-H_2O$</th><th>Mono-isotopic mass (Da)</th><th></th></tr>
<tr><td rowspan="6">a</td><td rowspan="6">Wild type</td><td rowspan="6">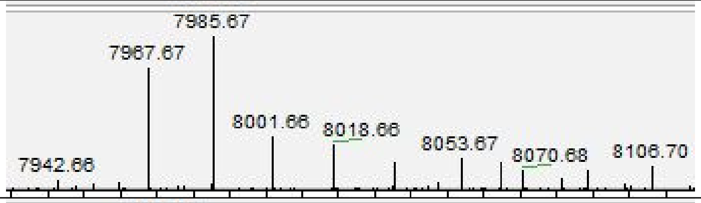
</td><td>0</td><td>8057,70</td><td rowspan="6"></td></tr>
<tr><td>1</td><td>8039,69</td></tr>
<tr><td>2</td><td>8021,68</td></tr>
<tr><td>3</td><td>8003,67</td></tr>
<tr><td>4</td><td>7985,66</td></tr>
<tr><td>5</td><td>7967,65</td></tr>
<tr><td rowspan="6">b</td><td rowspan="6">D-5A</td><td rowspan="6">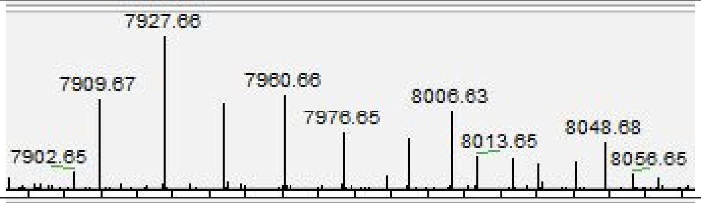
</td><td>0</td><td>7999,69</td><td rowspan="6"></td></tr>
<tr><td>1</td><td>7981,68</td></tr>
<tr><td>2</td><td>7963,67</td></tr>
<tr><td>3</td><td>7945,66</td></tr>
<tr><td>4</td><td>7927,65</td></tr>
<tr><td>5</td><td>7909,64</td></tr>
<tr><td rowspan="6">c</td><td rowspan="6">E-3A</td><td rowspan="6">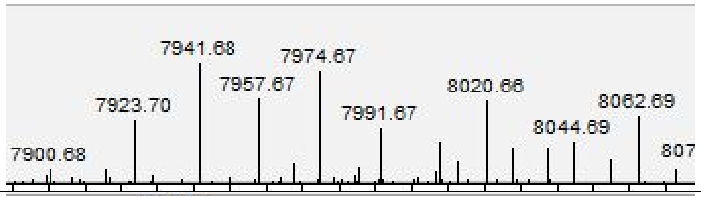
</td><td>0</td><td>8013,71</td><td rowspan="6"></td></tr>
<tr><td>1</td><td>7995,70</td></tr>
<tr><td>2</td><td>7977,69</td></tr>
<tr><td>3</td><td>7959,68</td></tr>
<tr><td>4</td><td>7941,67</td></tr>
<tr><td>5</td><td>7923,66</td></tr>
<tr><td rowspan="6">d</td><td rowspan="6">D-5N</td><td rowspan="6">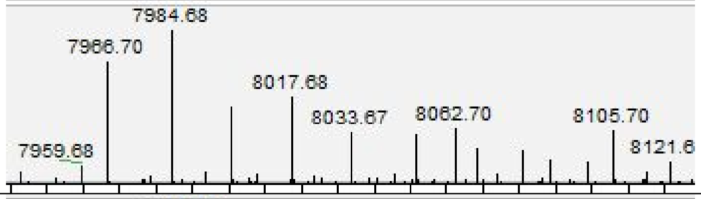
</td><td>0</td><td>8056,71</td><td rowspan="6"></td></tr>
<tr><td>1</td><td>8038,70</td></tr>
<tr><td>2</td><td>8020,69</td></tr>
<tr><td>3</td><td>8002,68</td></tr>
<tr><td>4</td><td>7984,67</td></tr>
<tr><td>5</td><td>7966,66</td></tr>
<tr><td rowspan="6">e</td><td rowspan="6">E-3Q</td><td rowspan="6">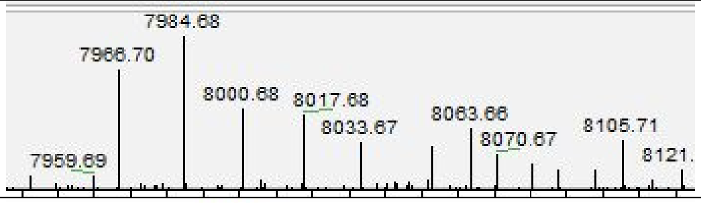
</td><td>0</td><td>8056,71</td><td rowspan="6"></td></tr>
<tr><td>1</td><td>8038,70</td></tr>
<tr><td>2</td><td>8020,69</td></tr>
<tr><td>3</td><td>8002,68</td></tr>
<tr><td>4</td><td>7984,67</td></tr>
<tr><td>5</td><td>7966,66</td></tr>
<tr><td rowspan="6">f</td><td rowspan="6">E-3A D5A</td><td rowspan="6">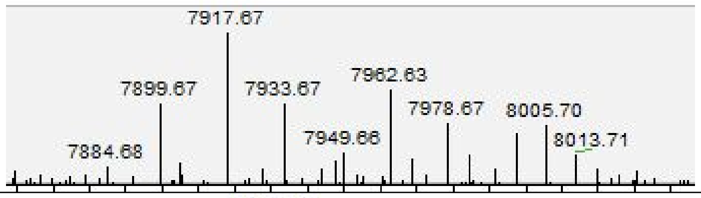
</td><td>0</td><td>7955,70</td><td rowspan="6">Mostly -3 H_2O - $2H^+$, with trace amounts of -4 H_2O.</td></tr>
<tr><td>1</td><td>7937,69</td></tr>
<tr><td>2</td><td>7919,68</td></tr>
<tr><td>3</td><td>7901,67</td></tr>
<tr><td>4</td><td>7883,66</td></tr>
<tr><td>5</td><td>7865,65</td></tr>
<tr><td rowspan="6">g</td><td rowspan="6">E-3Q D5N</td><td rowspan="6">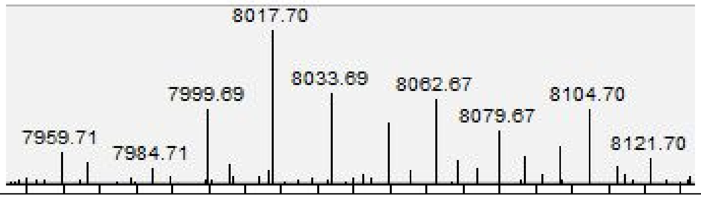
</td><td>0</td><td>8055,73</td><td rowspan="6">Mostly -3 H_2O - $2H^+$, with trace amounts of -4 H_2O.</td></tr>
<tr><td>1</td><td>8037,72</td></tr>
<tr><td>2</td><td>8019,71</td></tr>
<tr><td>3</td><td>8001,70</td></tr>
<tr><td>4</td><td>7983,69</td></tr>
<tr><td>5</td><td>7965,68</td></tr>
</table>

	Mutant	Spectrum	- H_2O	Mono-isotopic mass (Da)	
h	E-3K	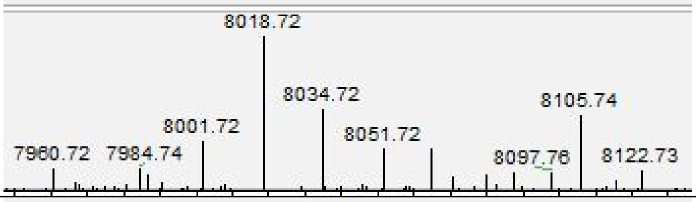	0	8056,75	-5 H_2O undetectable, but antimicrobial activity shows its presence
			1	8038,74	
			2	8020,73	
			3	8002,72	
			4	7984,71	
			5	7966,70	
i	D-5K	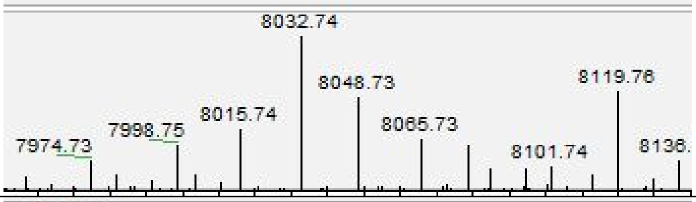	0	8070,77	-5 H_2O undetectable, but antimicrobial activity shows its presence
			1	8052,76	
			2	8034,75	
			3	8016,73	
			4	7998,72	
			5	7980,71	
j	AGDMEA	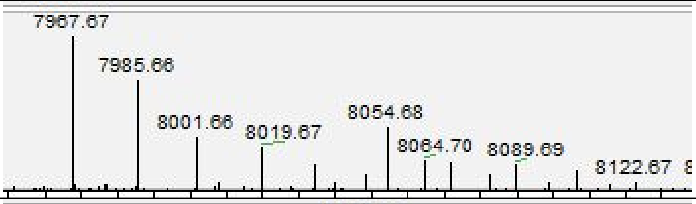	0	8057,70	Fully dehydrated, but at least one ring is not formed.
			1	8039,69	
			2	8021,68	
			3	8003,67	
			4	7985,66	
			5	7967,65	
k	DMEAAA	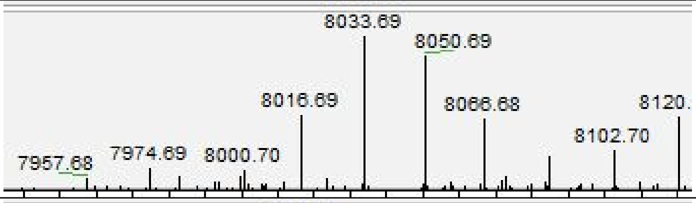	0	8071,71	At most -3 H_2O, with trace amounts of -4 H_2O.
			1	8053,70	
			2	8035,69	
			3	8017,68	
			4	7999,67	
			5	7981,66	
l	ΔDMEA	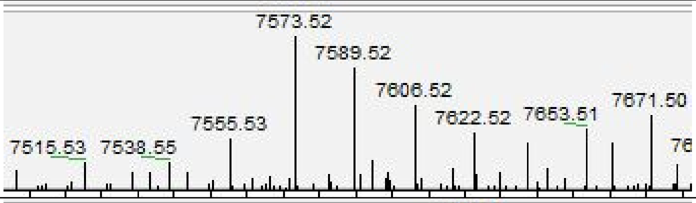	0	7611,55	Mostly -3 H_2O - $2H^+$, with trace amounts of -4 H_2O.
			1	7593,54	
			2	7575,53	
			3	7557,52	
			4	7539,51	
			5	7521,50	
m	ΔDMEAA	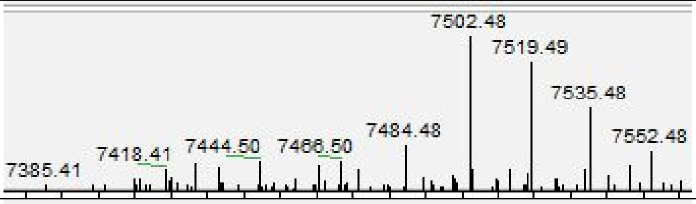	0	7540,51	Mostly -3 H_2O - $2H^+$
			1	7522,50	
			2	7504,49	
			3	7486,48	
			4	7468,47	
			5	7450,46	
n	ΔGDMEAA	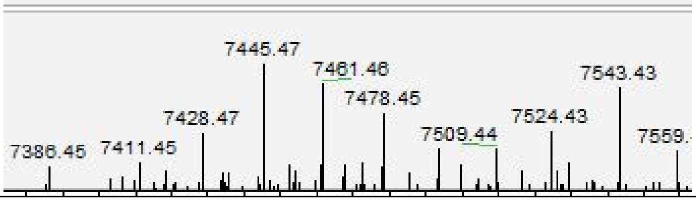	0	7483,49	Mostly -3 H_2O - $2H^+$, with trace amounts of -4 H_2O.
			1	7465,48	
			2	7447,47	
			3	7429,46	
			4	7411,45	
			5	7393,44	

	Mutant	Spectrum	$-H_2O$	Mono-isotopic mass (Da)	
o	ΔA-1	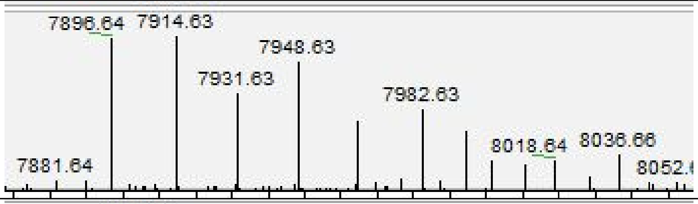	0<br>1<br>2<br>3<br>4<br>5	7986,66<br>7968,65<br>7950,64<br>7932,63<br>7914,62<br>7896,61	Fully dehydrated, but at least one ring is not formed.
p	ΔG-6	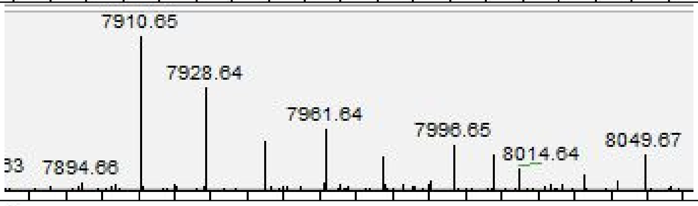	0<br>1<br>2<br>3<br>4<br>5	8000,68<br>7982,67<br>7964,66<br>7946,65<br>7928,63<br>7910,62	Production and modification as good as wild type
q	ΔK-11	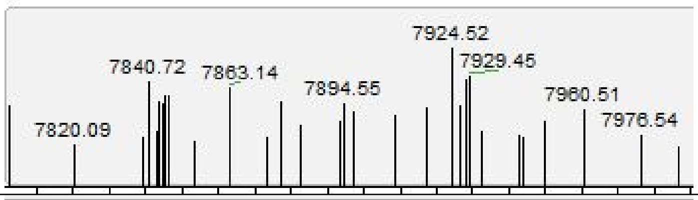	0<br>1<br>2<br>3<br>4<br>5	7929,60<br>7911,59<br>7893,58<br>7875,57<br>7857,56<br>7839,55	Poor production and modification efficiency
r	ΔE-16	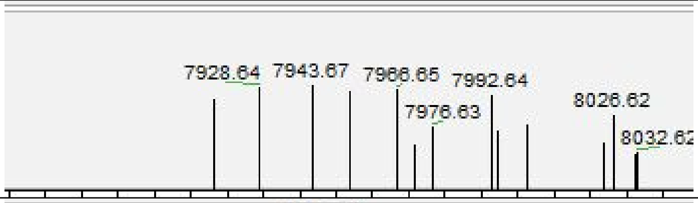	0<br>1<br>2<br>3<br>4<br>5	7928,66<br>7910,65<br>7892,63<br>7874,62<br>7856,61<br>7838,60	Very poor production. No modification
s	ΔF-21	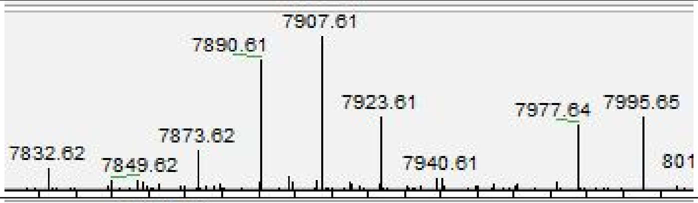	0<br>1<br>2<br>3<br>4<br>5	7910,63<br>7892,62<br>7874,61<br>7856,60<br>7838,59<br>7820,58	Poor production and modification efficiency
t	K-11A	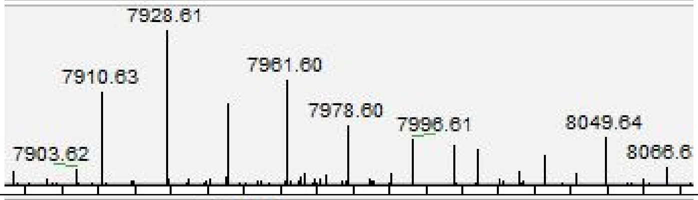	0<br>1<br>2<br>3<br>4<br>5	8000,64<br>7982,63<br>7964,62<br>7946,61<br>7928,60<br>7910,59	Production and modification almost as good as wild type
u	E-16A	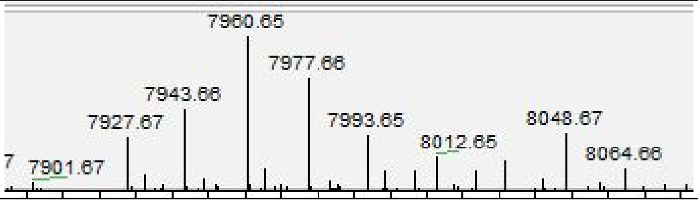	0<br>1<br>2<br>3<br>4<br>5	7999,69<br>7981,68<br>7963,67<br>7945,66<br>7927,65<br>7909,64	$-5\ H_2O$ undetectable, but antimicrobial activity shows its presence

	Mutant	Spectrum	$-H_2O$	Mono-isotopic mass (Da)	
V	F-21A	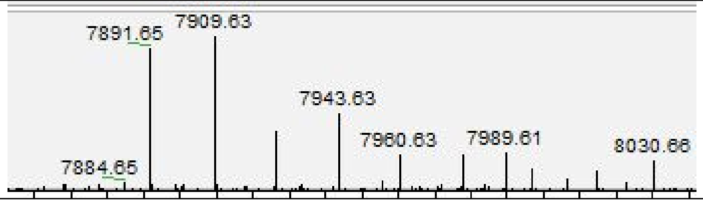	0	7981,67	
			1	7963,66	
			2	7945,65	
			3	7927,64	
			4	7909,62	
			5	7891,61	

S4. Antimicrobial activity tests of all mutants

To assure maximum resolution of the activity tests, all mutants were first applied in high amounts, applying yields from 16 mL of culture digested by AprE-His (plate 1-3). As a control, an AprE-His digested mixed yield from three wild type expressions was applied in an equivalent of 4 mL of culture on all plates. Also 225 ng of nisin was spotted as a positive control. Next, the mutants showing an activity high enough to be limited by diffusion were tested again (plate 4), this time using half the amount (8 mL of culture equivalent). Finally, the compounds in plate five were spotted in equal amounts as the control, namely 4 mL equivalent of expression culture.

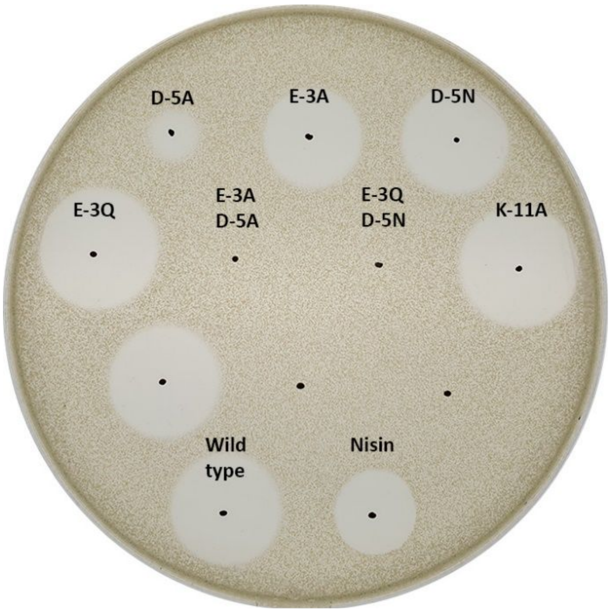

Plate 1: Mutants spotted **4:1** compared to wild type control.

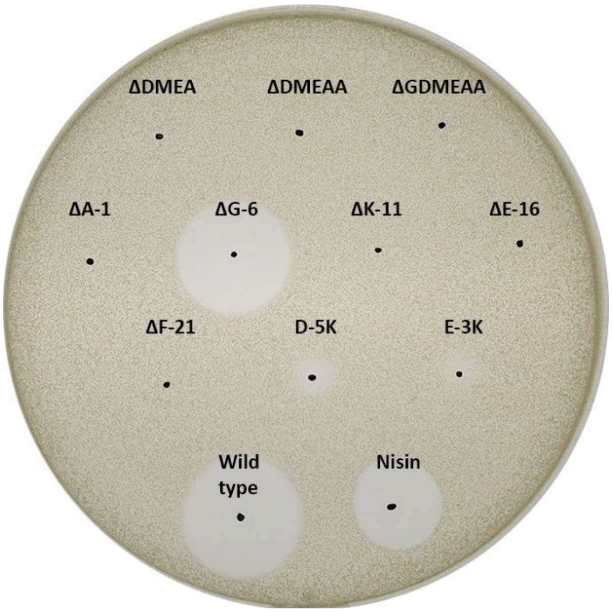

Plate 2: Mutants spotted **4:1** compared to wild type control.

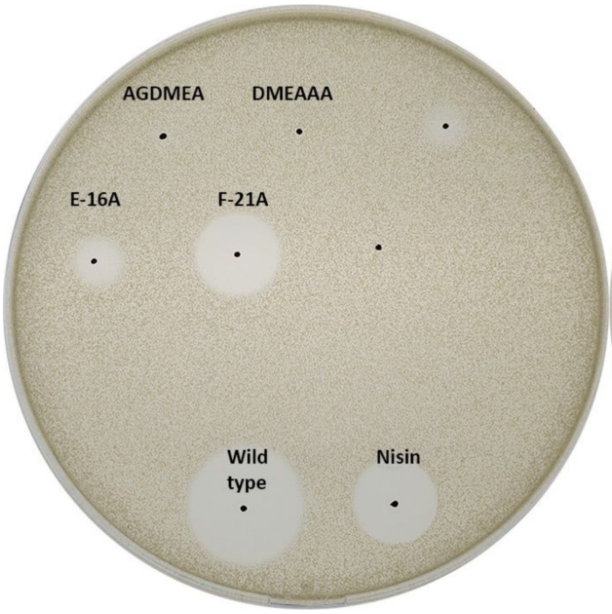

Plate 3: Mutants spotted **4:1** compared to wild type control.

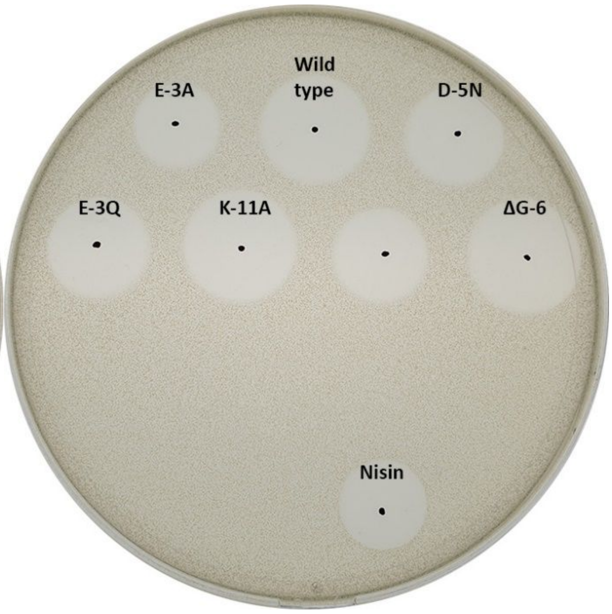

Plate 4: Mutants spotted **2:1** compared to wild type control.

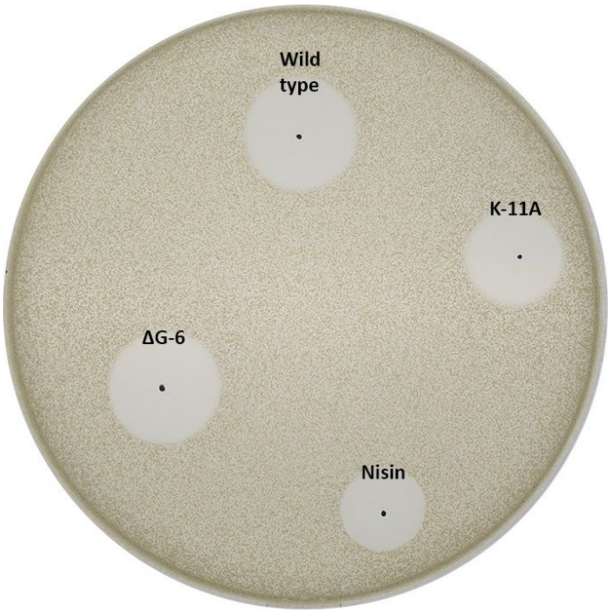

Plate 5: Mutants spotted **1:1** compared to wild type control.

To determine the relative activity, the area of each of the tested mutants was compared with that of the wild type control, which was set to 1. For mutants tested in more than one volume, the halo from the smallest spotted volume was used. The difference in surface area compared to the wild type control was directly translated to a fraction of the wild type activity. Based on this fraction, an activity rating of 0 to 5 was given based on an exponential scale best representing the differences in activity between the tested mutants (See table below).

Relative antimicrobial activity

	Mutant	Plate	Halo Diameter (cm)	Halo Area	Amount Ratio	Activity per ratio	Fraction of activity	>0	>0,09375	>0,1875	>0,375	>0,75
c	D-5A	1	1,98	3,08	4	0,77	0,05	+			**(of wildtype activity)**	
f	E-3A D-5A	1	0,00	0,00	4	0,00	0,00					
g	E-3Q N-5A	1	0,00	0,00	4	0,00	0,00					
a	***wild type***	1	4,55	16,26	1	16,26	1,00					
l	ΔDMEA	2	0,00	0,00	4	0,00	0,00					
m	ΔDMEAA	2	0,00	0,00	4	0,00	0,00					
n	ΔGDMEAA	2	0,00	0,00	4	0,00	0,00					
o	ΔA-1	2	0,00	0,00	4	0,00	0,00					
q	ΔK-11	2	0,00	0,00	4	0,00	0,00					
r	ΔE-16	2	0,00	0,00	4	0,00	0,00					
s	ΔF-21	2	0,00	0,00	4	0,00	0,00					
i	D-5K	2	1,10	0,95	4	0,24	0,01	+				
h	E-3K	2	0,75	0,44	4	0,11	0,01	+				
a	***wild type***	2	4,91	18,93	1	18,93	1,00					
j	AGDMEA	3	0,00	0,00	4	0,00	0,00					
k	DMEAAA	3	0,00	0,00	4	0,00	0,00					
u	E-16A	3	1,50	1,77	4	0,44	0,03	+				
v	F-21A	3	3,26	8,35	4	2,09	0,12	+	+			
a	***wild type***	3	4,70	17,35	1	17,35	1,00					
b	E-3A	4	3,46	9,40	2	4,70	0,30	+	+	+		
e	D-5N	4	3,85	11,64	2	5,82	0,37	+	+	+		
d	E-3Q	4	3,89	11,88	2	5,94	0,38	+	+	+	+	
a	***wild type***	4	4,48	15,76	1	15,76	1,00					
t	K-11A	5	3,58	10,07	1	10,07	0,70	+	+	+	+	
p	ΔG-6	5	4,35	14,86	1	14,86	1,03	+	+	+	+	+
a	***wild type***	5	4,28	14,39	1	14,39	1,00					

S5. Spectra of AprE cleaved mutants

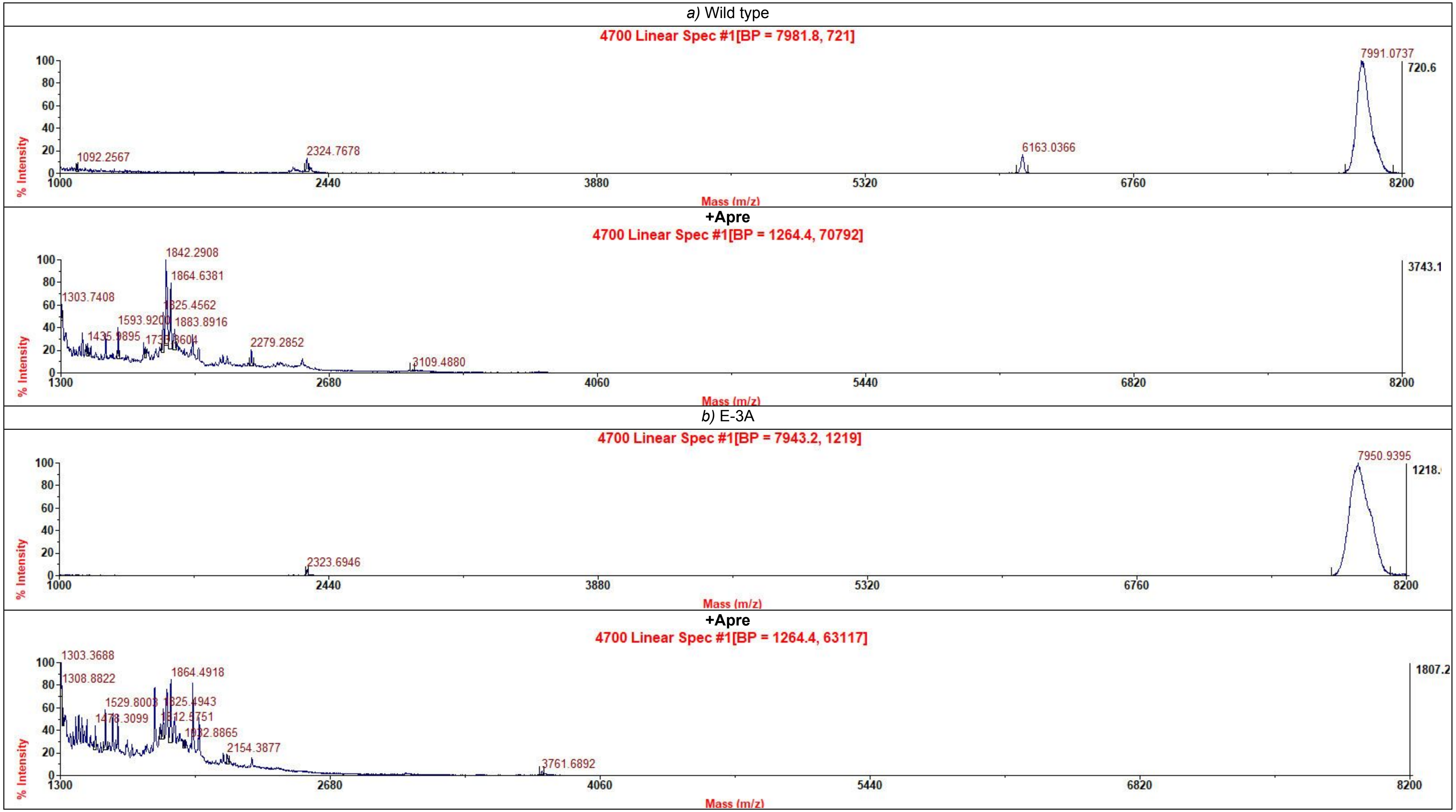

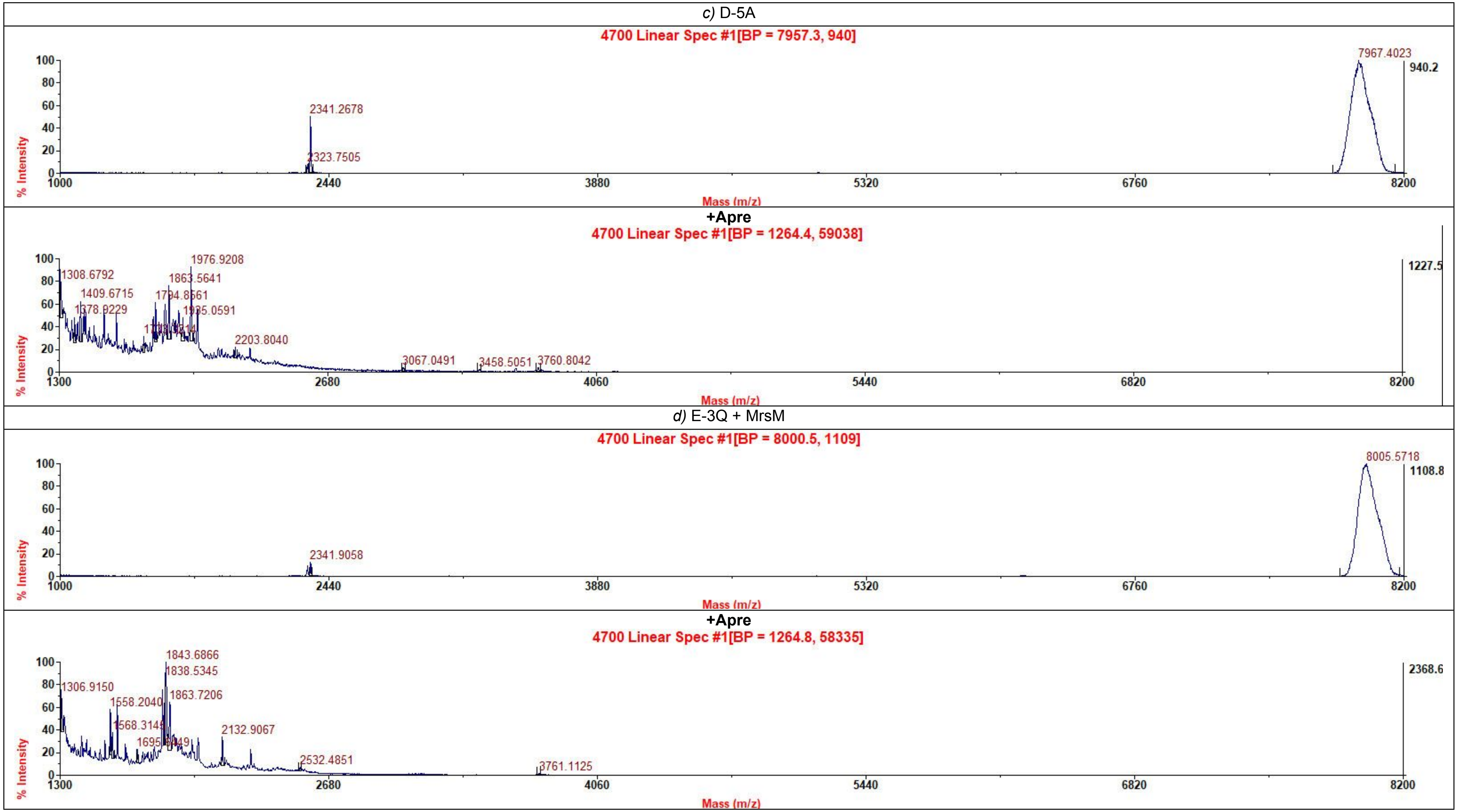

c) D-5A
4700 Linear Spec #1[BP = 7957.3, 940]
% Intensity
Mass (m/z)
7967.4023
940.2
2341.2678
2323.7505
1000
2440
3880
5320
6760
8200
+Apre
4700 Linear Spec #1[BP = 1264.4, 59038]
1976.9208
1308.6792
1863.5641
1409.6715
1794.8561
1378.9229
1935.0591
2203.8040
3067.0491
3458.5051
3760.8042
1227.5
1300
2680
4060
5440
6820
8200
d) E-3Q + MrsM
4700 Linear Spec #1[BP = 8000.5, 1109]
8005.5718
1108.8
2341.9058
+Apre
4700 Linear Spec #1[BP = 1264.8, 58335]
1843.6866
1838.5345
1306.9150
1558.2040
1863.7206
1568.3146
2132.9067
2532.4851
3761.1125
2368.6

e) D-5N

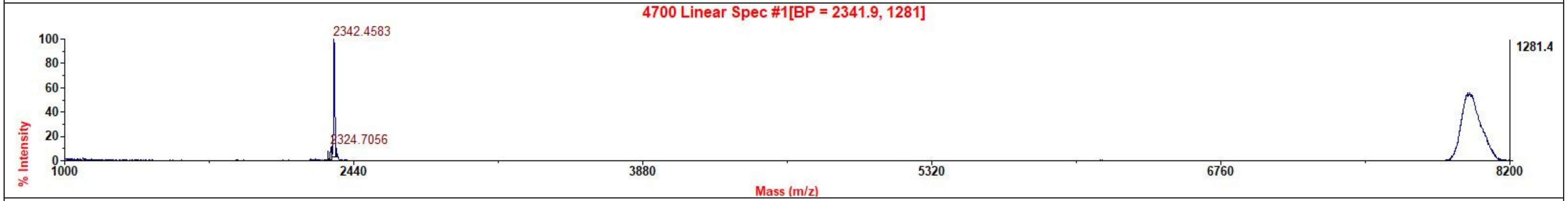

+Apre

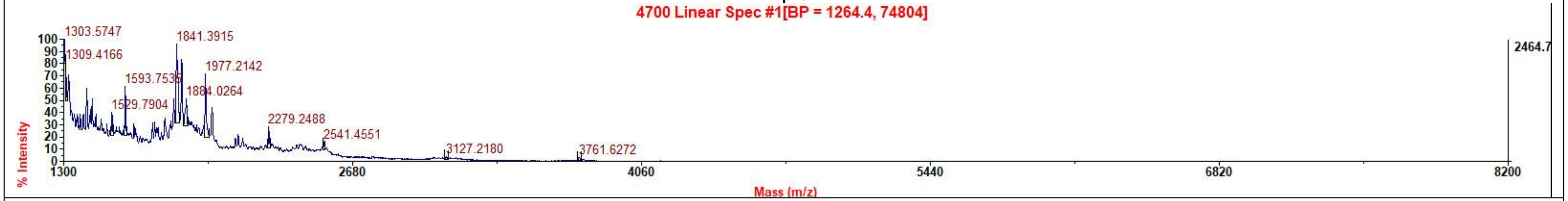

f) E-3A D-5A

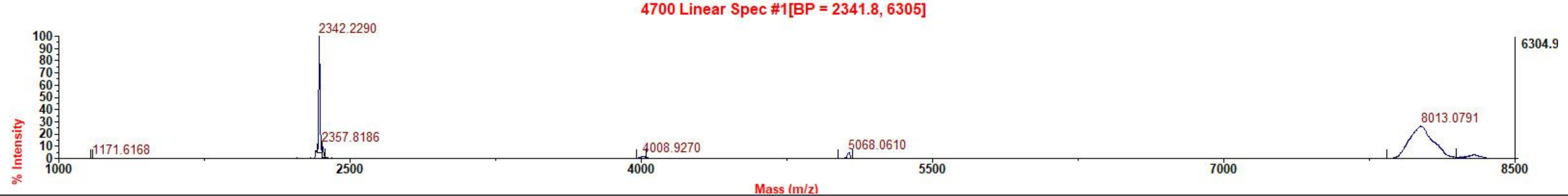

+Apre

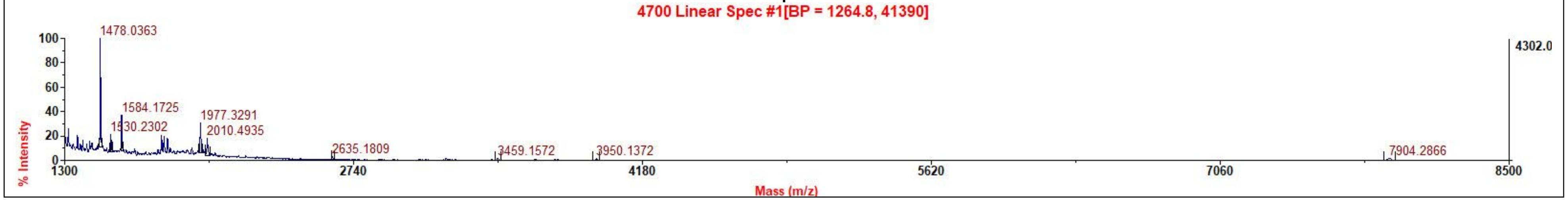

g) E-3Q N-5A

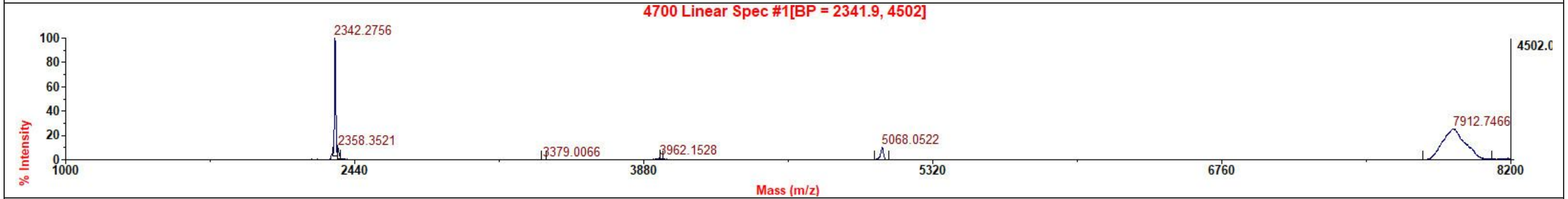

+Apre

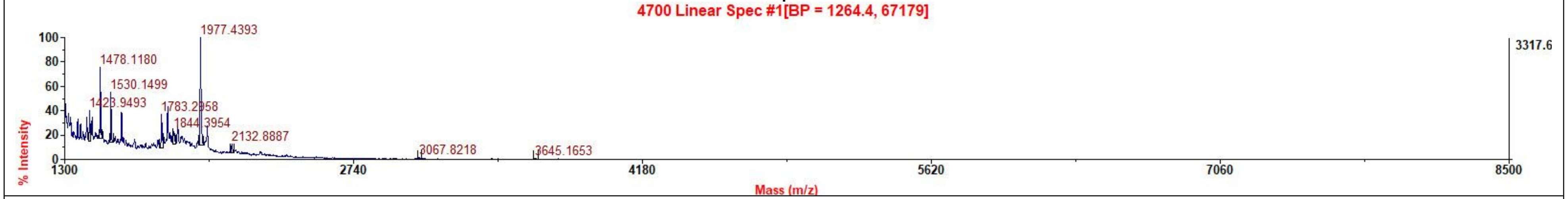

h) E-3K

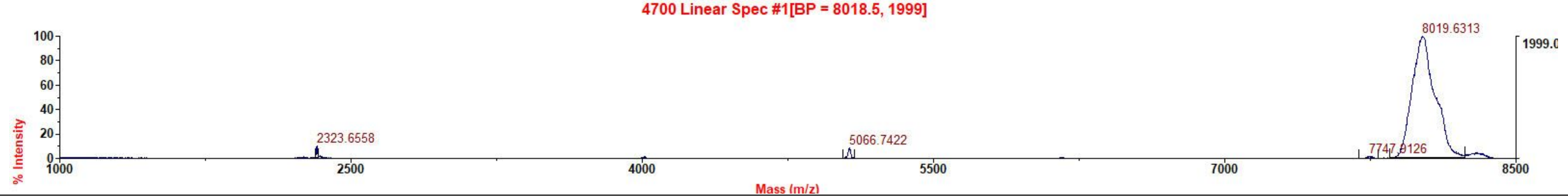

+Apre

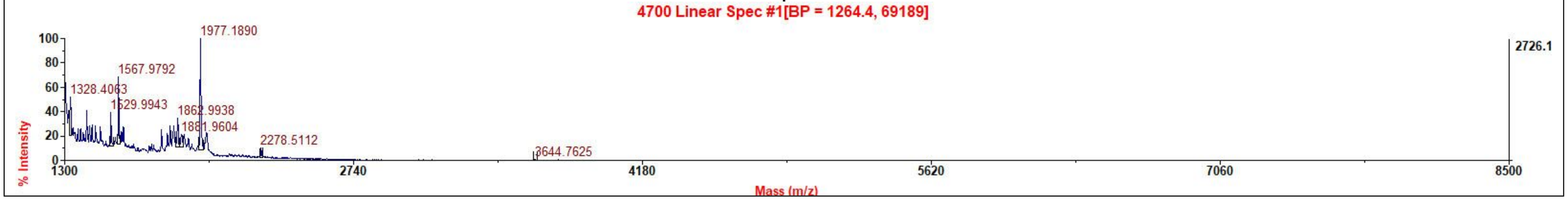

i) D-5K

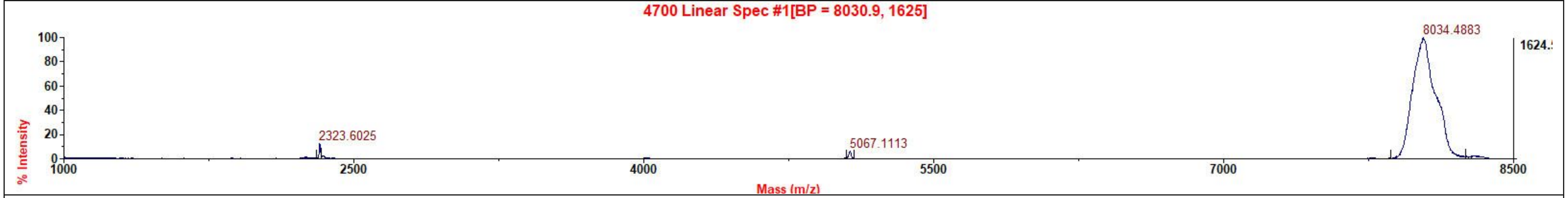

+Apre

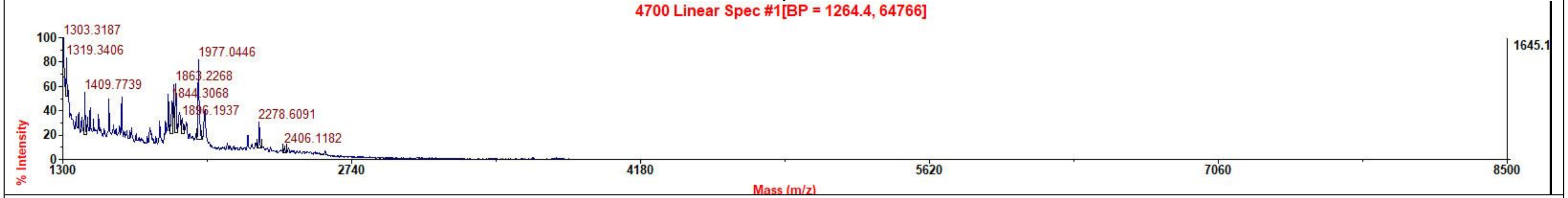

j) AGDMEA

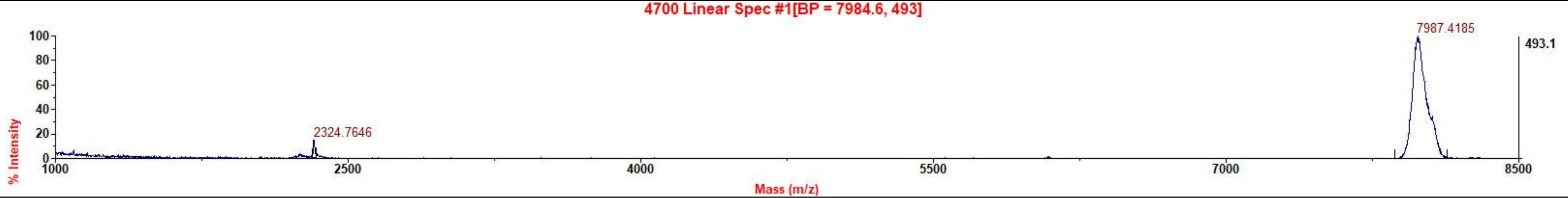

+Apre

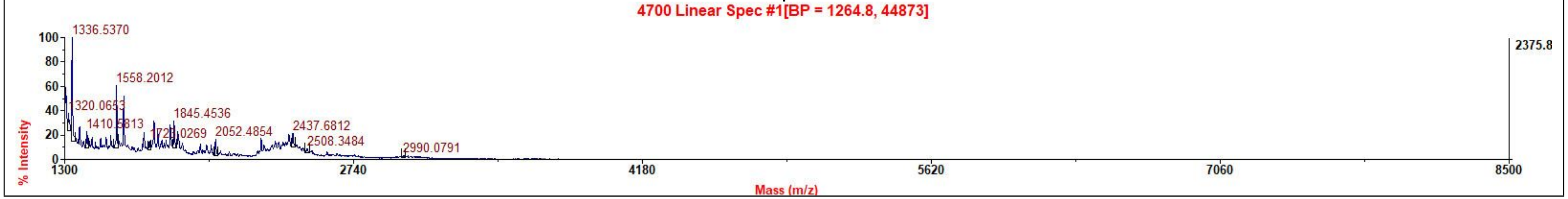

k) DMEAAA

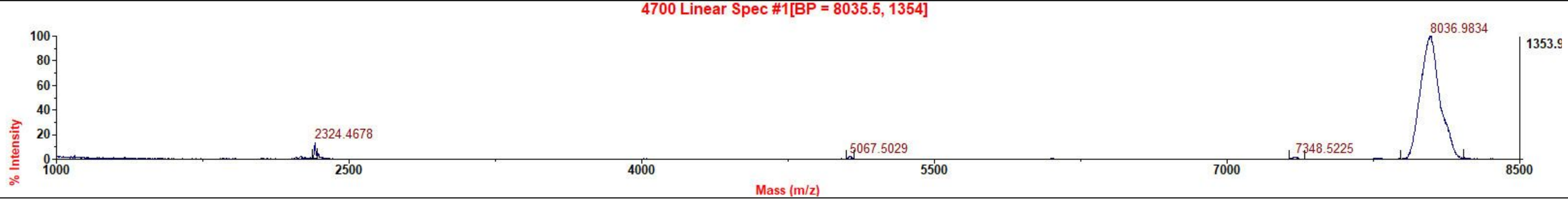

+Apre

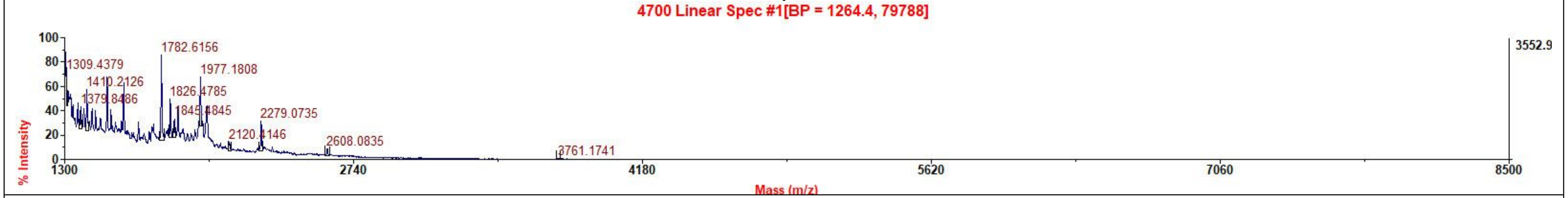

l) ΔDMEA

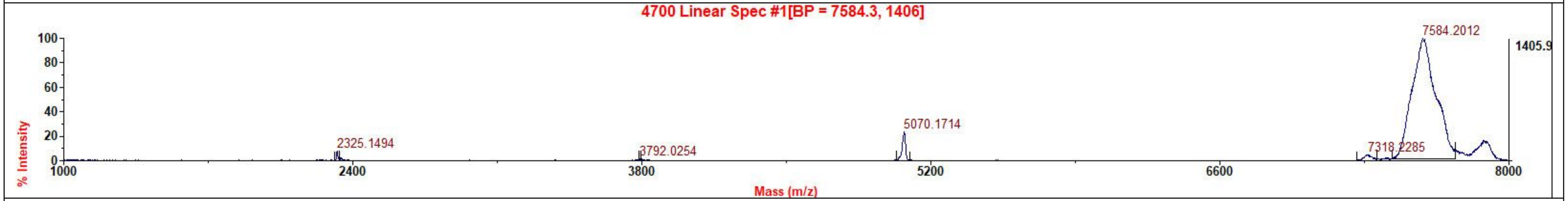

+Apre

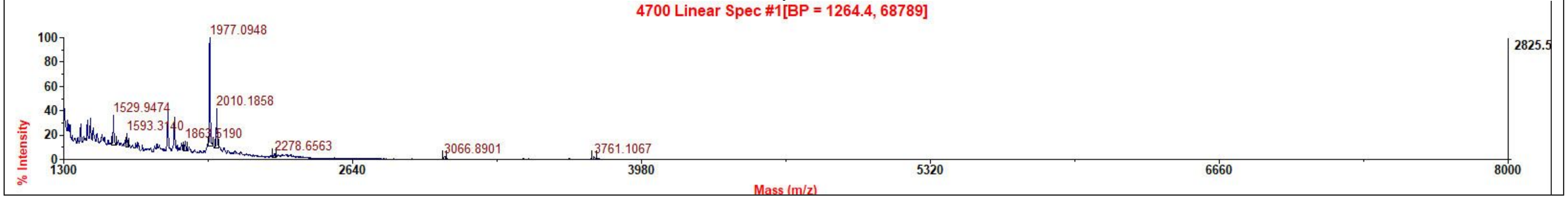

m) ΔDMEAA

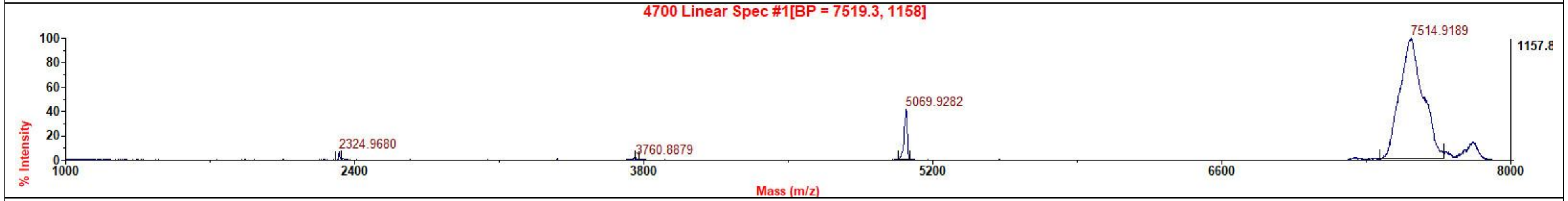

+Apre

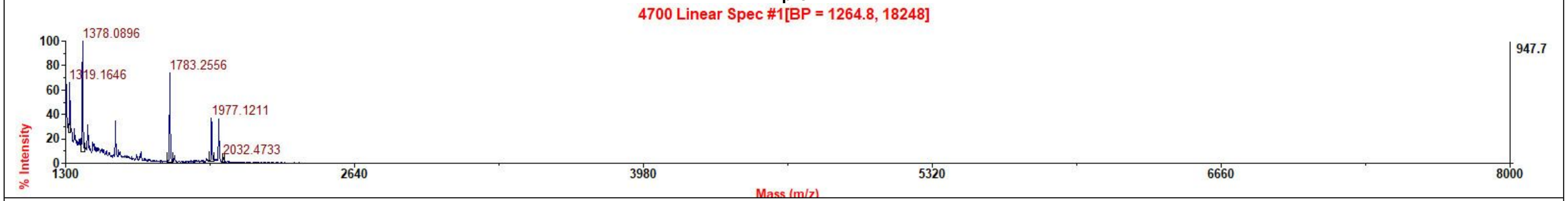

n) ΔGDMEAA

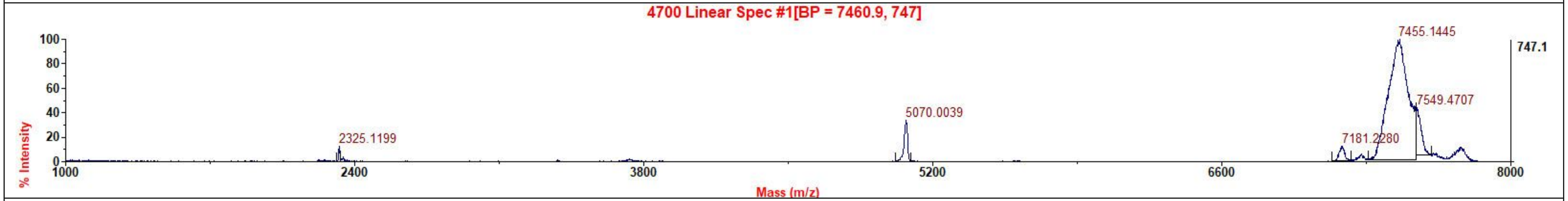

+Apre

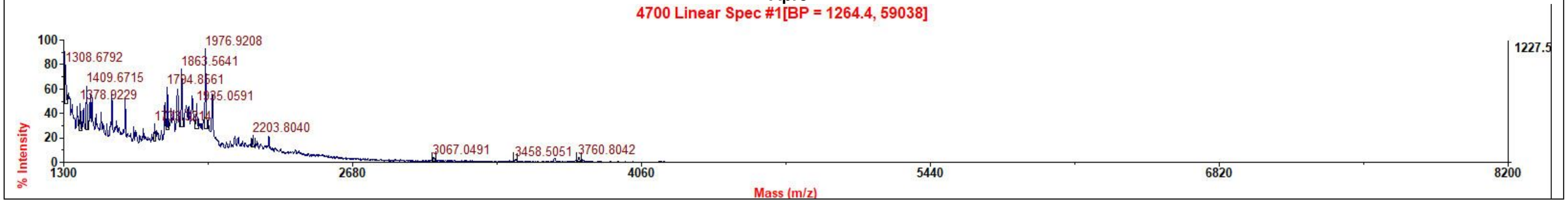

o) ΔA-1

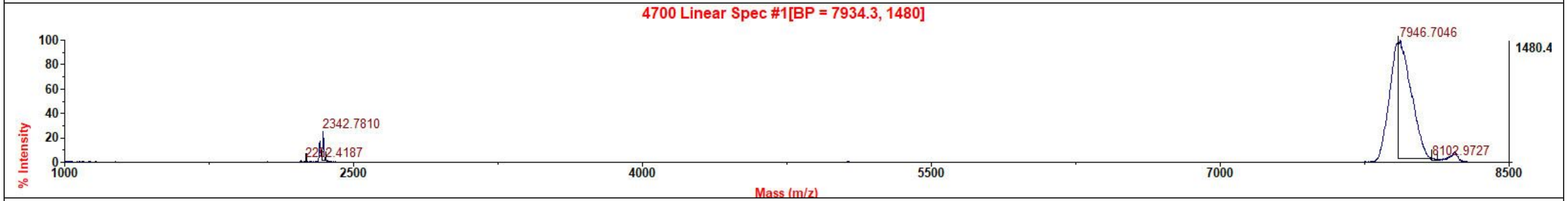

+Apre

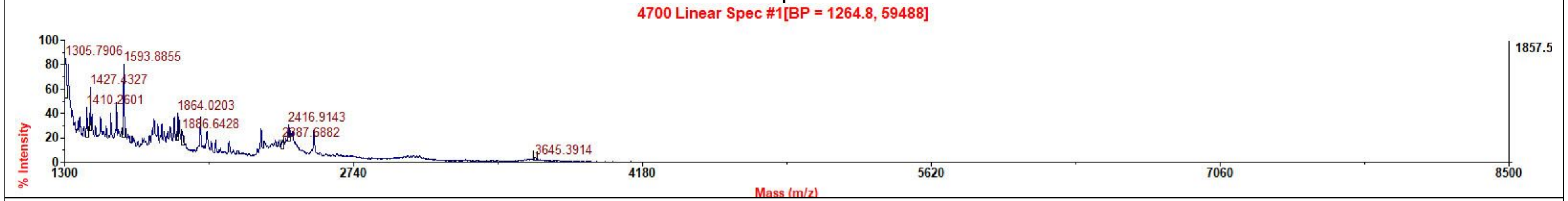

p) ΔG-6

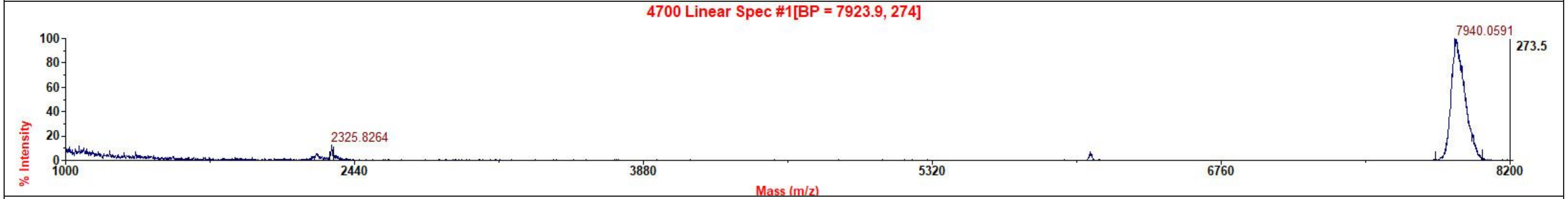

+Apre

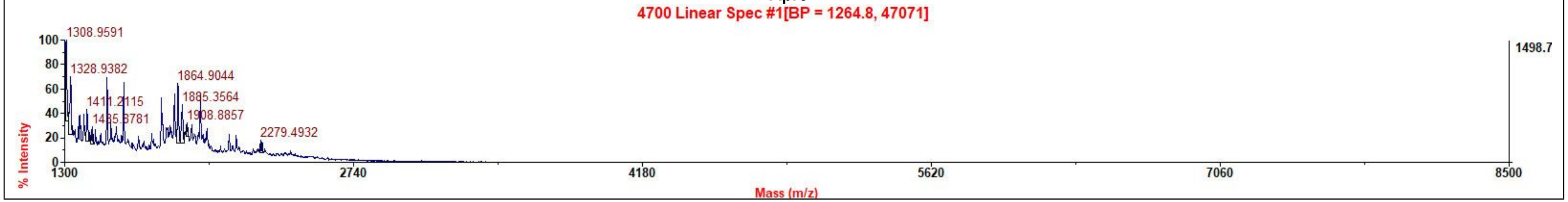

q) ΔK-11

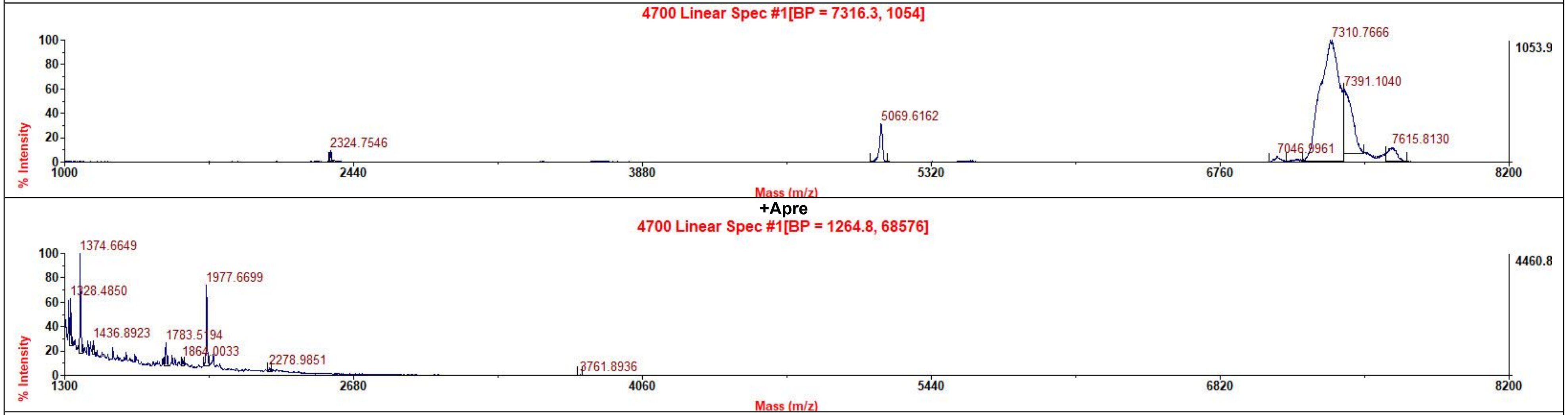
4700 Linear Spec #1[BP = 7316.3, 1054]
7310.7666
7391.1040
5069.6162
2324.7546
7046.9961
7615.8130
1053.9
% Intensity
Mass (m/z)
+Apre
4700 Linear Spec #1[BP = 1264.8, 68576]
1374.6649
1977.6699
1328.4850
1436.8923
1783.5194
1864.0033
2278.9851
3761.8936
4460.8
% Intensity
Mass (m/z)

r) ΔE-16

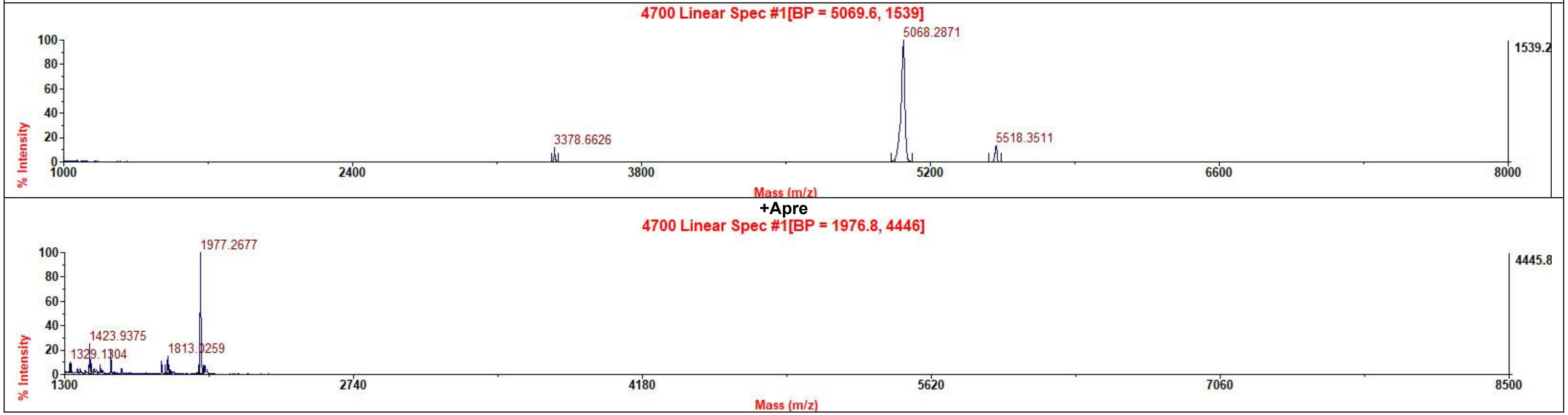
4700 Linear Spec #1[BP = 5069.6, 1539]
5068.2871
3378.6626
5518.3511
1539.2
% Intensity
Mass (m/z)
+Apre
4700 Linear Spec #1[BP = 1976.8, 4446]
1977.2677
1423.9375
1329.1304
1813.0259
4445.8
% Intensity
Mass (m/z)

s) ΔF-21

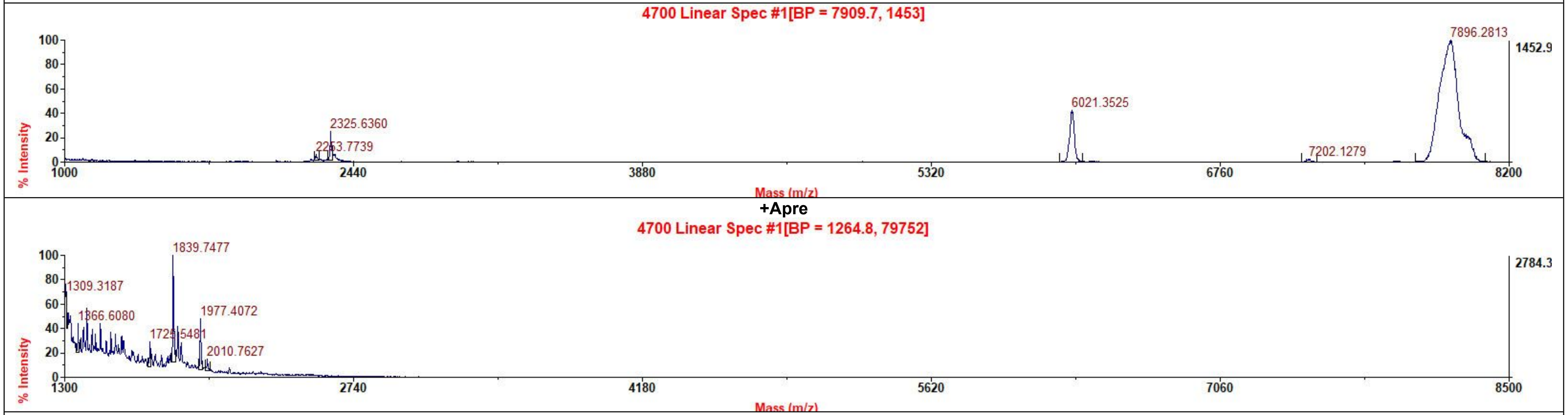

4700 Linear Spec #1[BP = 7909.7, 1453]
7896.2813
1452.9
6021.3525
2325.6360
2253.7739
7202.1279
% Intensity
Mass (m/z)
+Apre
4700 Linear Spec #1[BP = 1264.8, 79752]
1839.7477
2784.3
1309.3187
1366.6080
1977.4072
1725.5481
2010.7627

t) K-11A

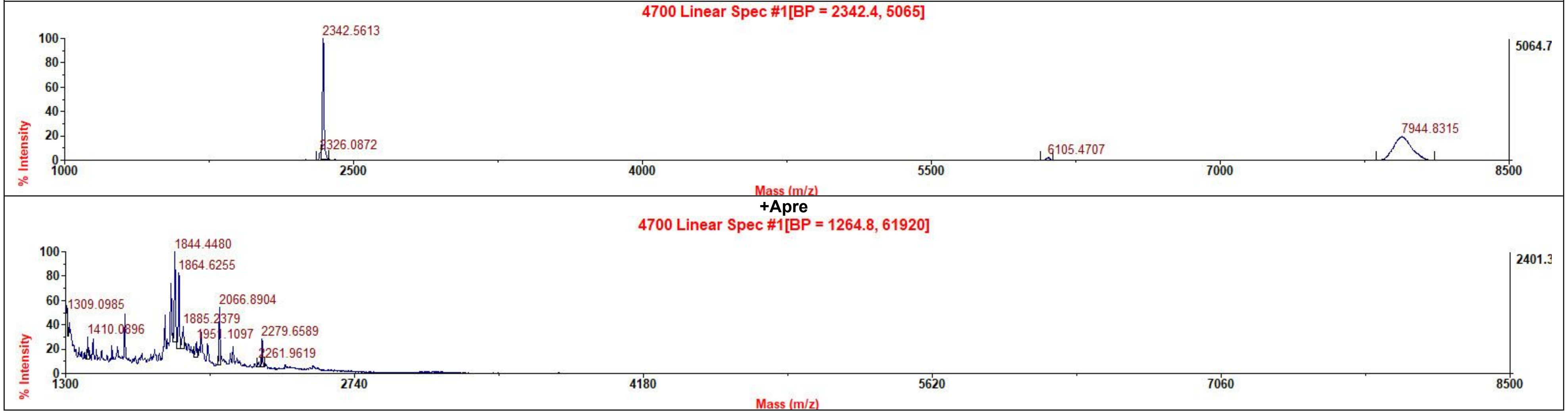

4700 Linear Spec #1[BP = 2342.4, 5065]
2342.5613
5064.7
2326.0872
6105.4707
7944.8315
% Intensity
Mass (m/z)
+Apre
4700 Linear Spec #1[BP = 1264.8, 61920]
1844.4480
1864.6255
1309.0985
2066.8904
1885.2379
1410.0896
1951.1097
2279.6589
2261.9619

u) E-16A

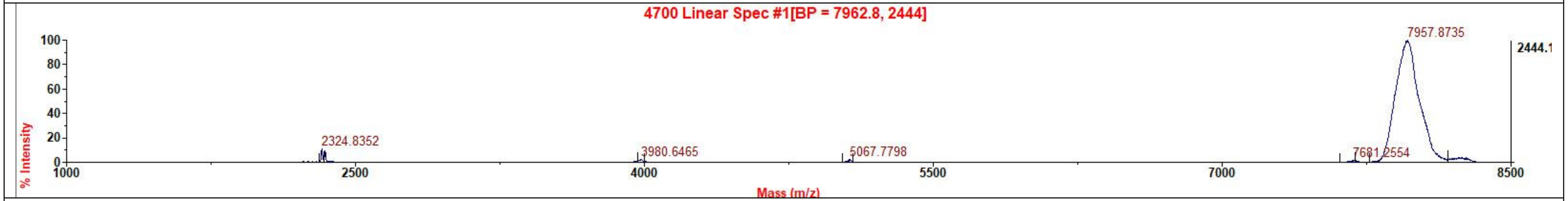

+Apre

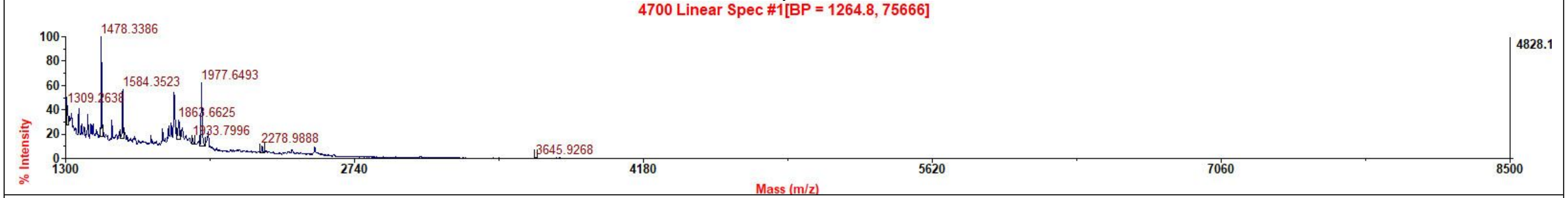

v) F-21A

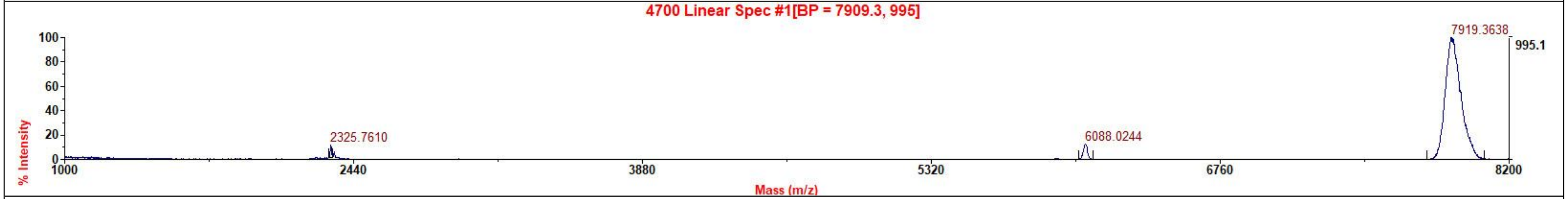

+Apre

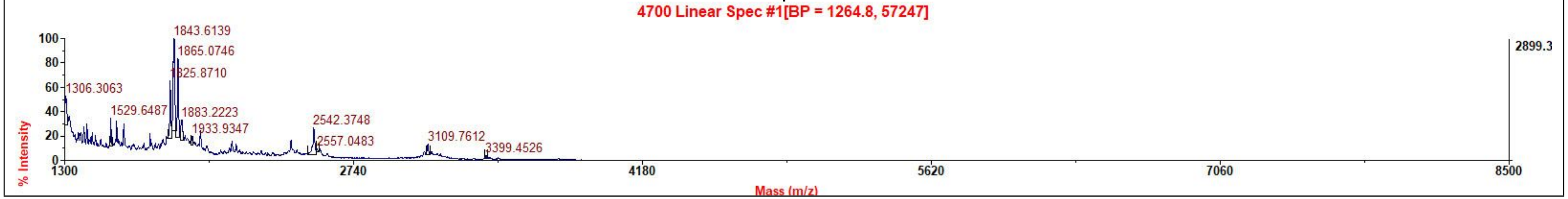

S6. Comparison of AprE digestion patterns wild type, AGDMEA and ΔA-1

If the lack of activity detected for the AGDMEA and ΔA-1 mutants was caused by AprE no longer being able to cleave the mersacidin leader, peaks larger than the mersacidin mass (1826 Da) should be visible by MALDI-TOF analysis. While the detected peaks that have a higher mass than mersacidin, have a much higher intensity than those in the wild type, these peaks are fragments of the leader peptide not containing mersacidin. Of both tested mutants, the fragments containing mersacidin and up to five amino acids from the leader peptide, should differ one alanine residue in mass. These differences are not detected. In fact, Masses resembling the peak of mersacidin are detected in both the AGDMEAA and ΔA-1 mutant's spectra. This shows that AprE can still cleave before the core peptide sequence. The fact that no activity is detected from these cleaved peptides confirms the results from the free cysteine essay, that not all rings have been formed.

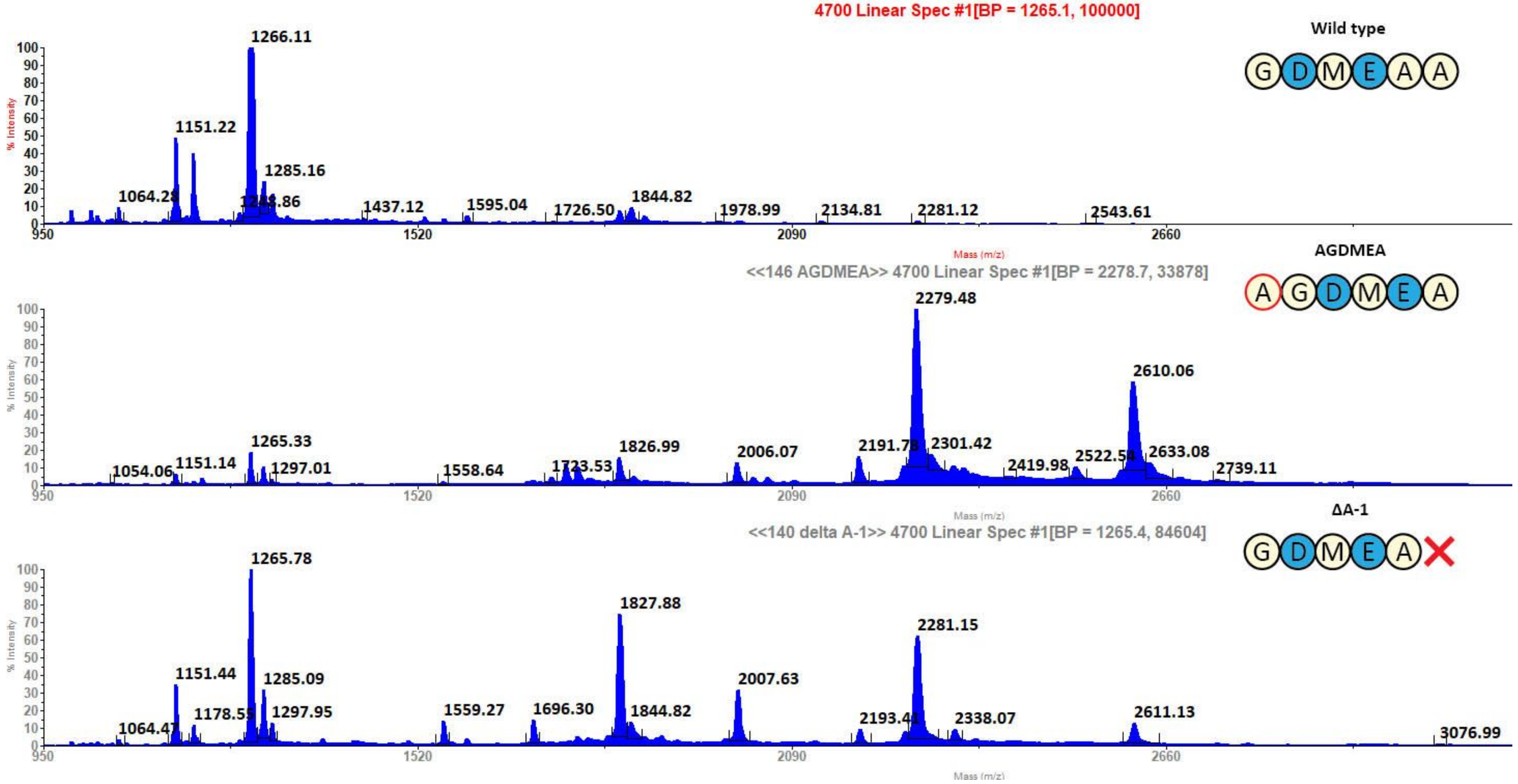

S7. Table of theoretical average masses

Theoretical average masses of all leader mutants in Dalton

		Unmodified	$-CO_2$	$-CO_2$ -1 H_2O	$-CO_2$ -2 H_2O	$-CO_2$ -3 H_2O	$-CO_2$ -4 H_2O	$-CO_2$ -5 H_2O
a	**Wild type (His6-MrsA)**	8108,99	8062,99	8044,97	8026,96	8008,94	7990,93	7972,91
b	pACYC E-3A + MrsM	8050,96	8004,95	7986,94	7968,92	7950,91	7932,89	7914,88
c	pACYC D-5A + MrsM	8064,98	8018,98	8000,96	7982,95	7964,93	7946,92	7928,90
d	pACYC E-3Q + MrsM	8108,01	8062,00	8043,99	8025,97	8007,96	7989,94	7971,93
e	pACYC D-5N + MrsM	8108,01	8062,00	8043,99	8025,97	8007,96	7989,94	7971,93
f	pACYC E-3A D-5A + MrsM	8006,95	7960,94	7942,93	7924,91	7906,90	7888,88	7870,87
g	pACYC E-3Q N-5A + MrsM	8107,02	8061,02	8043,00	8024,99	8006,97	7988,96	7970,94
h	pACYC E-3K + MrsM	8108,05	8062,05	8044,03	8026,01	8008,00	7989,98	7971,97
i	pACYC D-5K + MrsM	8122,08	8076,07	8058,06	8040,04	8022,03	8004,01	7986,00
j	pACYC AGDMEA + MrsM	8108,99	8062,99	8044,97	8026,96	8008,94	7990,93	7972,91
k	pACYC DMEAAA + MrsM	8123,02	8077,01	8059,00	8040,98	8022,97	8004,95	7986,94
l	pACYC ΔDMEA + MrsM	7662,52	7616,51	7598,50	7580,48	7562,46	7544,45	7526,43
m	pACYC ΔDMEAA + MrsM	7591,44	7545,43	7527,42	7509,40	7491,39	7473,37	7455,36
n	pACYC ΔGDMEAA + MrsM	7534,39	7488,38	7470,37	7452,35	7434,33	7416,32	7398,30
o	pACYC ΔA-1 + MrsM	8037,92	7991,91	7973,89	7955,88	7937,86	7919,85	7901,83
p	pACYC ΔG-6 + MrsM	8051,94	8005,94	7987,92	7969,90	7951,89	7933,87	7915,86
q	pACYC ΔK-11 + MrsM	7980,82	7934,81	7916,80	7898,78	7880,77	7862,75	7844,74
r	pACYC ΔE-16 + MrsM	7979,88	7933,87	7915,86	7897,84	7879,83	7861,81	7843,80
s	pACYC ΔF-21 + MrsM	7961,82	7915,81	7897,80	7879,78	7861,77	7843,75	7825,74
t	pACYC K-11A + MrsM	8051,90	8005,89	7987,88	7969,86	7951,85	7933,83	7915,82
u	pACYC E-16A + MrsM	8050,96	8004,95	7986,94	7968,92	7950,91	7932,89	7914,88
v	pACYC F-21A + MrsM	8032,90	7986,89	7968,88	7950,86	7932,85	7914,83	7896,82
Mersacidin leader +GDMEAA		6164,71						
Mersacidin leader -GDMEAA		5590,10						

S8. MrsT150-His cleavage analysis

The peptides, as verified by LC-MS mass analysis, were incubated with MrsT150-His, and analyzed by MALDI-TOF analysis. The large size prevents accurate depiction of the masses of individual dehydration states, but still gives a precise centroid mass. The mersacidin leader -GDMEAA theoretical average mass is 5590,10 Da (with the exception of variants where residues upstream of GDMEAA were mutated), which can be detected if MrsT150-His can process the leader mutant.

a) Wild type		
No MrsT	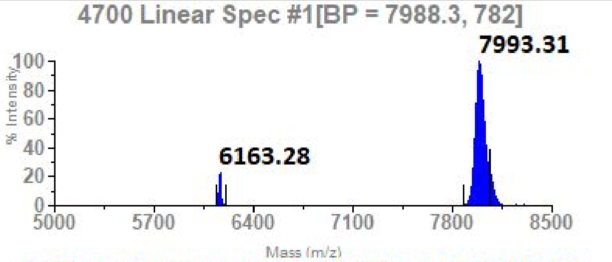	Is processed by MrsT150-His
+MrsT150-His	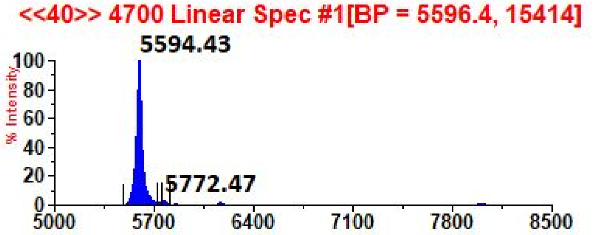	
b) E-3A		
No MrsT	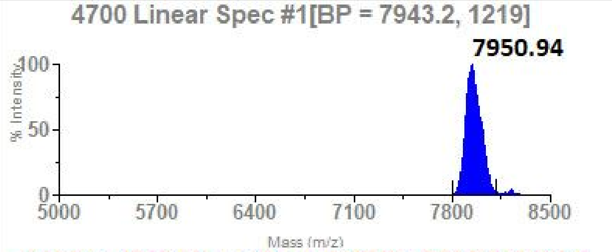	Is processed by MrsT150-His
+MrsT150-His	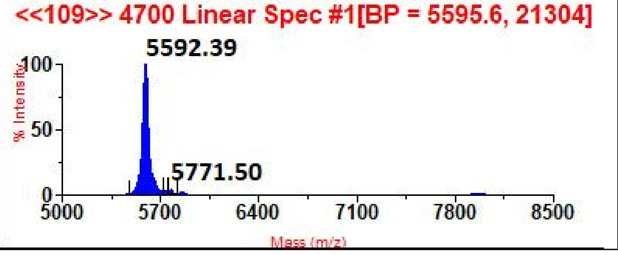	
c) D-5A		
No MrsT	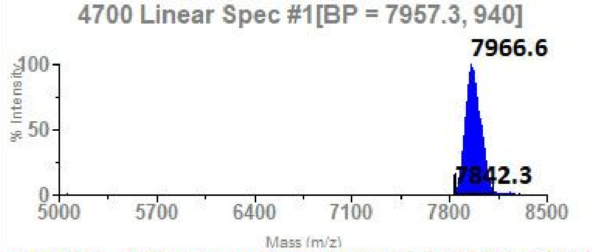	Is processed by MrsT150-His
+MrsT150-His	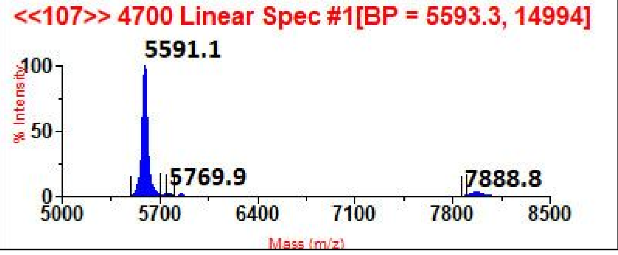	

d) D-5N		
No MrsT	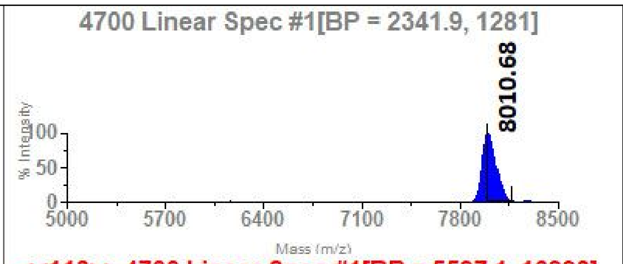	Is processed by MrsT150-His
+MrsT150-His	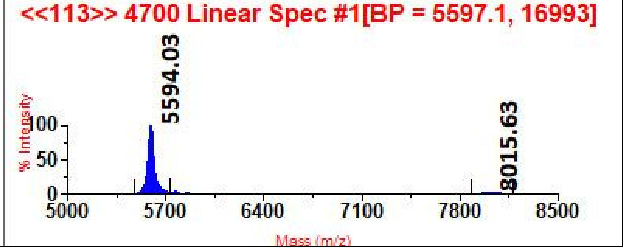	
e) E-3Q		
No MrsT	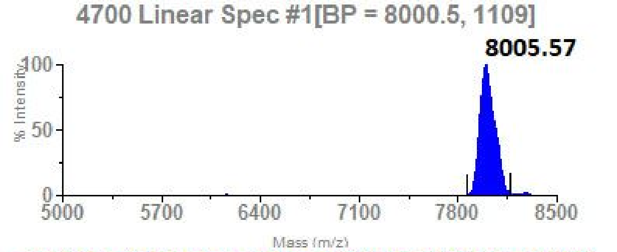	Is processed by MrsT150-His
+MrsT150-His	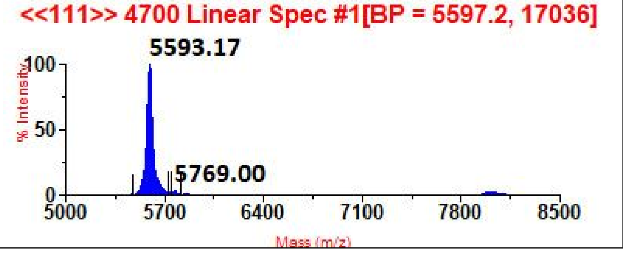	
f) E-3A D-5A		
No MrsT	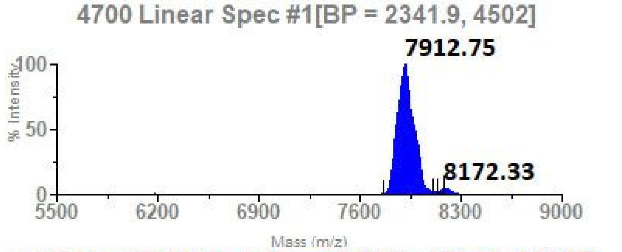	Is processed by MrsT150-His
+MrsT150-His	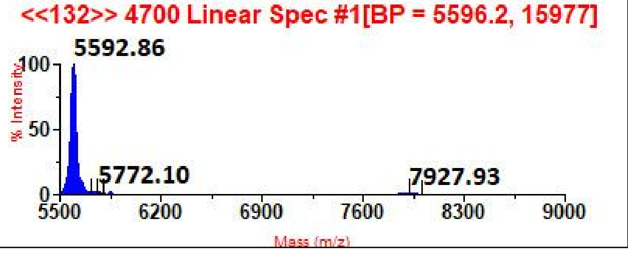	

g) E-3Q D-5N		
No MrsT	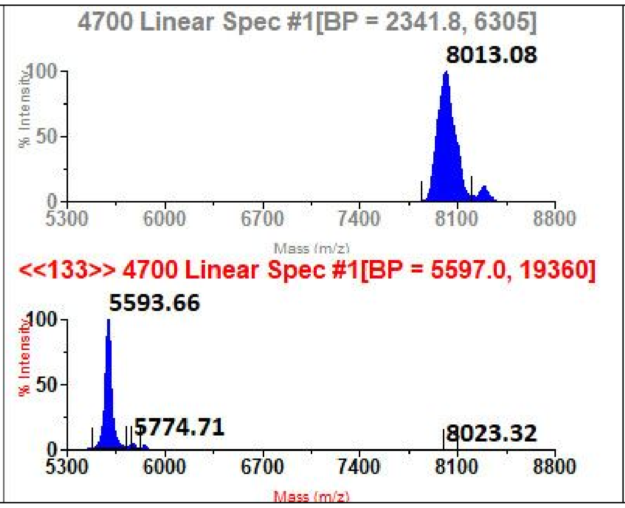	Is processed by MrsT150-His
+MrsT150-His		
***h*) E-3K**		
No MrsT	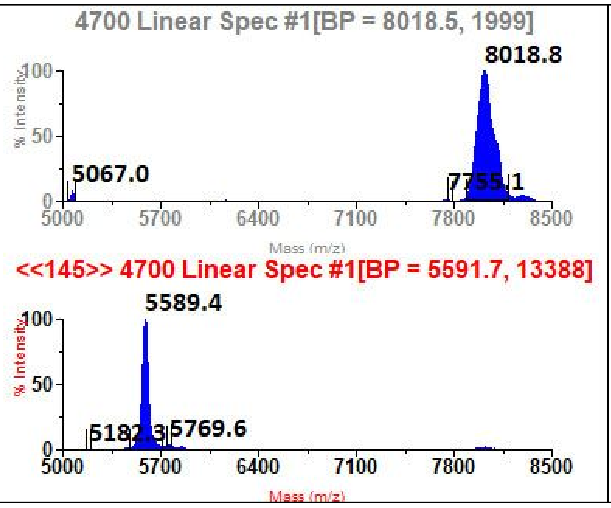	Is processed by MrsT150-His
+MrsT150-His		
***i*) D-5K**		
No MrsT	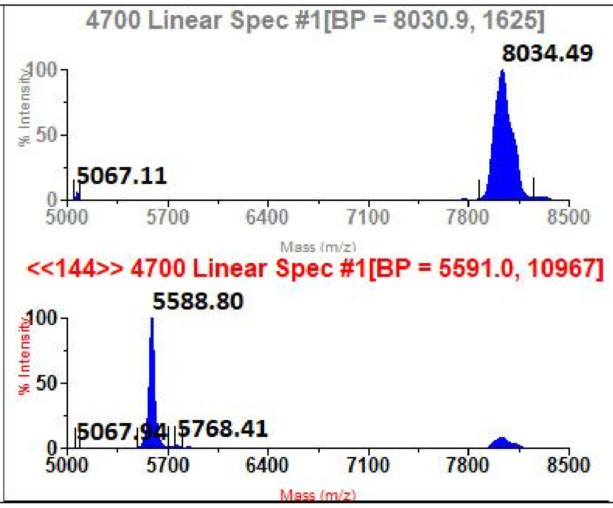	Is processed by MrsT150-His
+MrsT150-His		

j) AGDMEA		
No MrsT	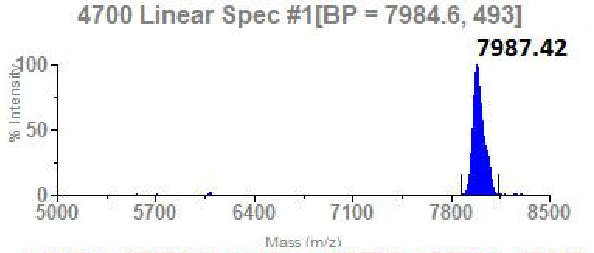	Is processed by MrsT150-His
+MrsT150-His	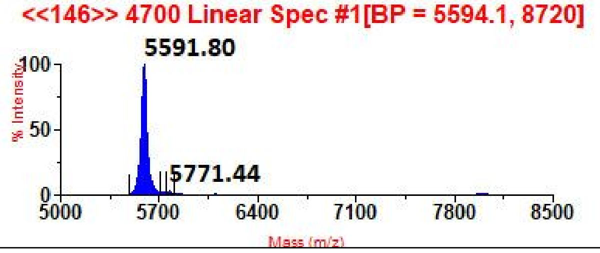	
k) DMEAAA		
No MrsT	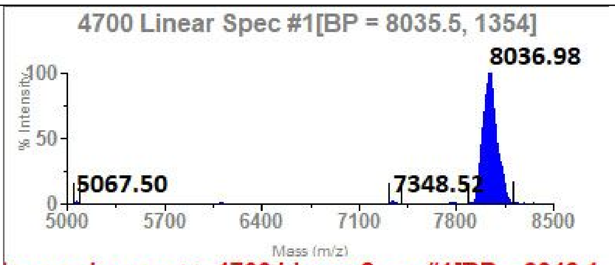	Is not processed well by MrsT150-His
+MrsT150-His	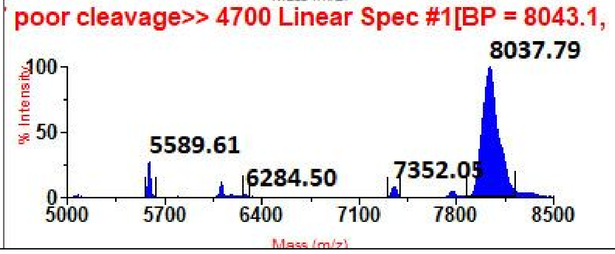	
l) ΔDMEA		
No MrsT	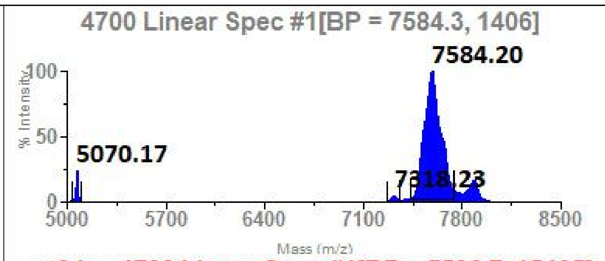	Is processed by MrsT150-His
+MrsT150-His	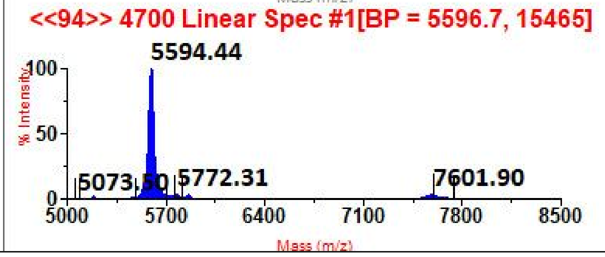	

m) ΔGDMEA		
No MrsT	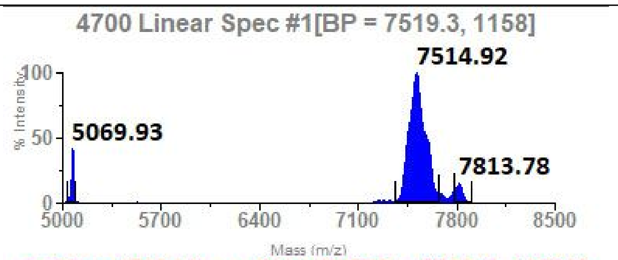	Is processed by MrsT150-His
+MrsT150-His	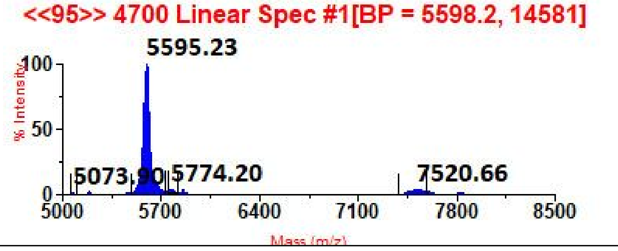	
n) ΔGDMEAA		
No MrsT	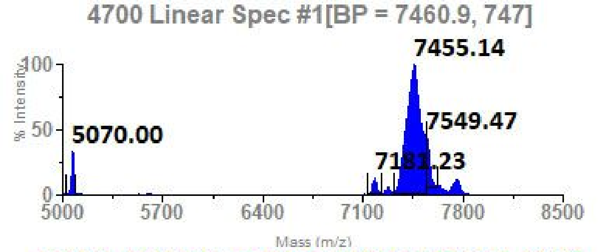	Is not processed by MrsT150-His
+MrsT150-His	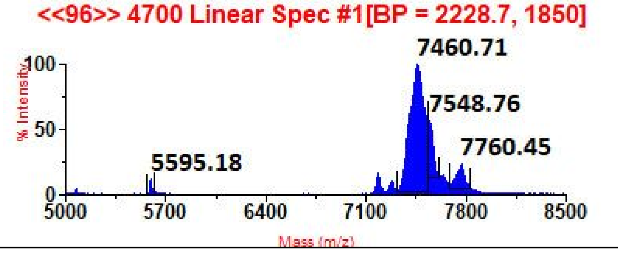	
o) ΔA-1		
No MrsT	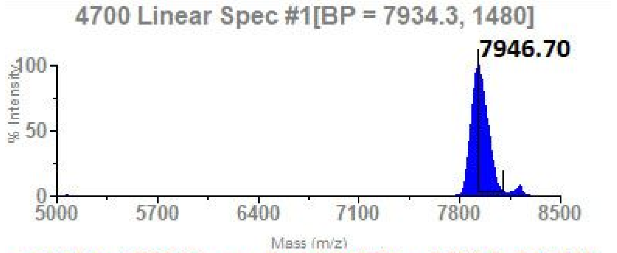	Is processed by MrsT150-His
+MrsT150-His	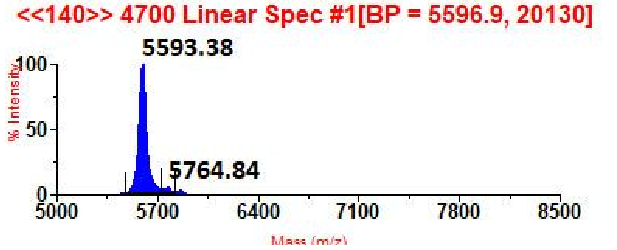	

<table>
<tr><td colspan="3">p) ΔG-6</td></tr>
<tr><td>No MrsT</td><td>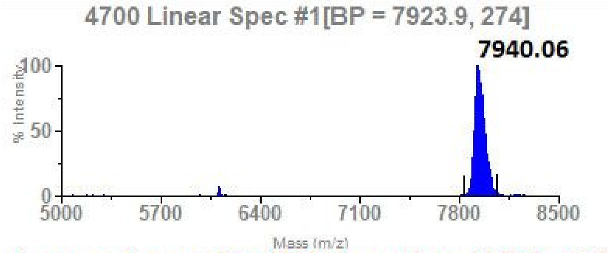
</td><td rowspan="2">Is not processed by MrsT150-His</td></tr>
<tr><td>+MrsT150-His</td><td>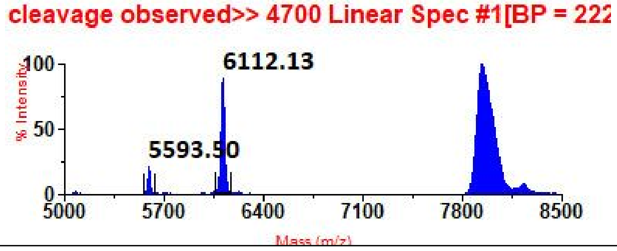
</td></tr>
<tr><td colspan="3">q) ΔK-11</td></tr>
<tr><td>No MrsT</td><td>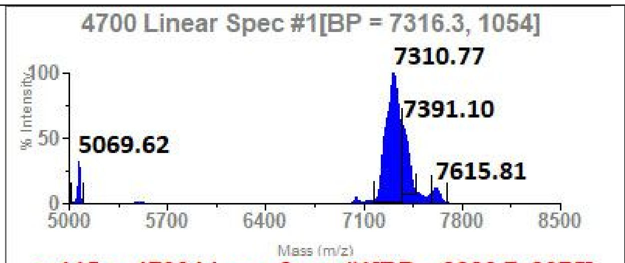
</td><td rowspan="2">Is not processed by MrsT150-His</td></tr>
<tr><td>+MrsT150-His</td><td>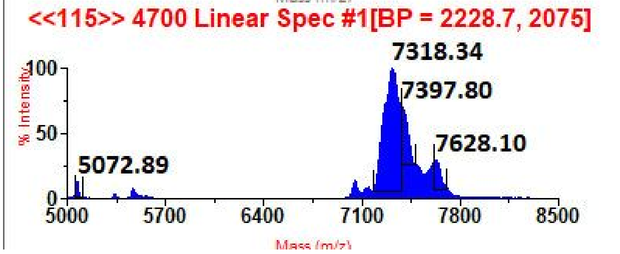
</td></tr>
<tr><td colspan="3">r) ΔE-16</td></tr>
<tr><td>No MrsT</td><td>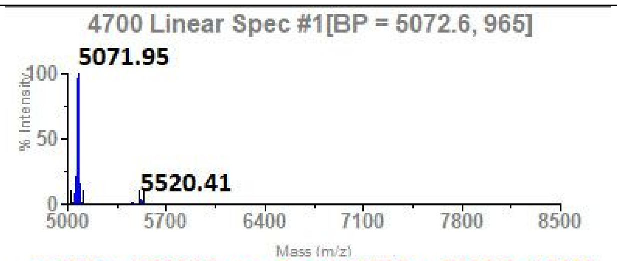
</td><td rowspan="2">Degraded prior to MrsT150-His digestion</td></tr>
<tr><td>+MrsT150-His</td><td>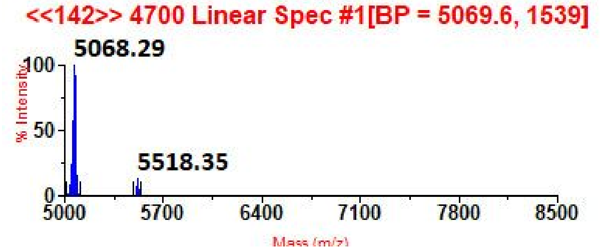
</td></tr>
</table>

s) ΔF-21		
No MrsT	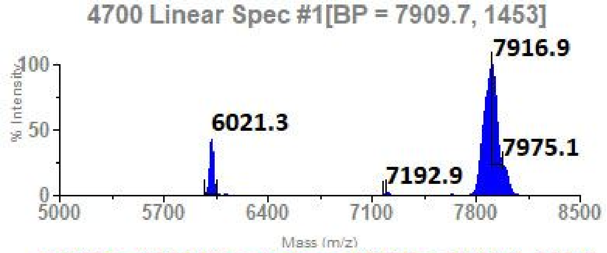	Is processed by MrsT150-His
+MrsT150-His	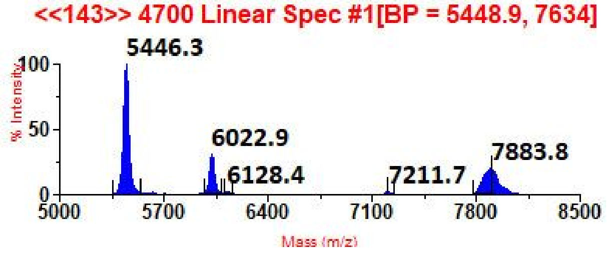	
t) K-11A		
No MrsT	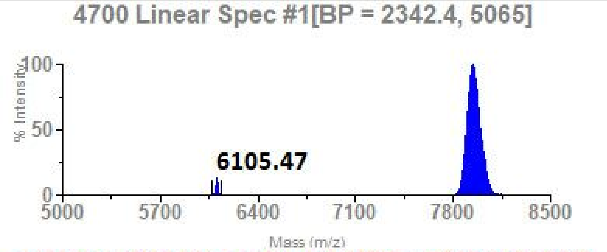	Is processed by MrsT150-His
+MrsT150-His	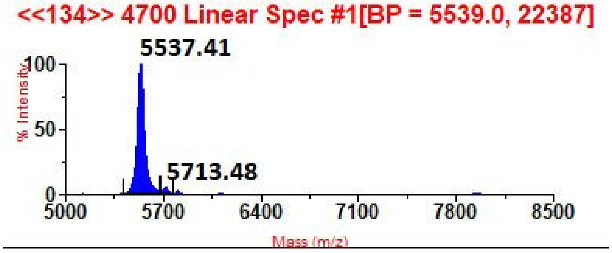	
u) E-16A		
No MrsT	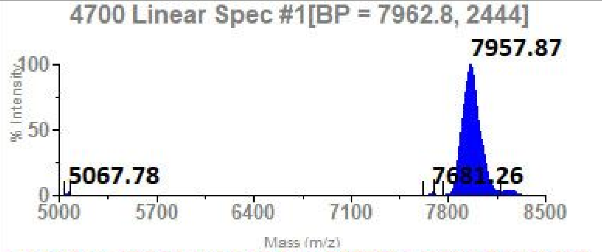	Is processed by MrsT150-His
+MrsT150-His	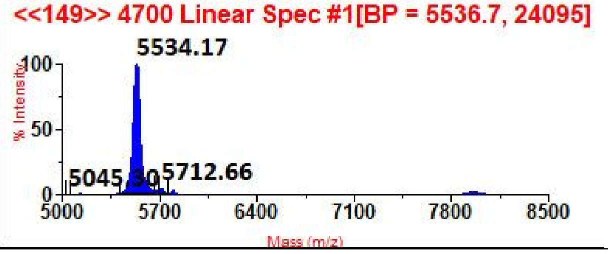	

<table>
<tr><td colspan="3">v) F-21A</td></tr>
<tr><td>No MrsT</td><td>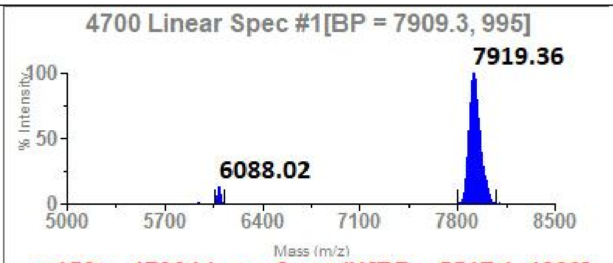
</td><td rowspan="2">Is processed by MrsT150-His</td></tr>
<tr><td>+MrsT150-His</td><td>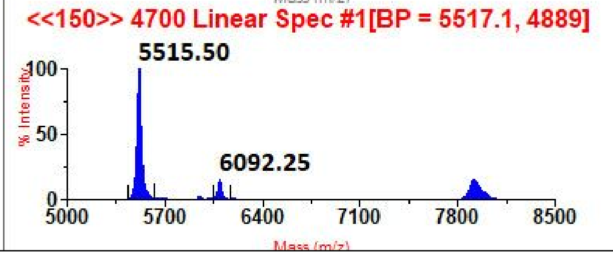
</td></tr>
<tr><td colspan="3">His6-mersacidin leader without core-peptide</td></tr>
<tr><td>No MrsT</td><td>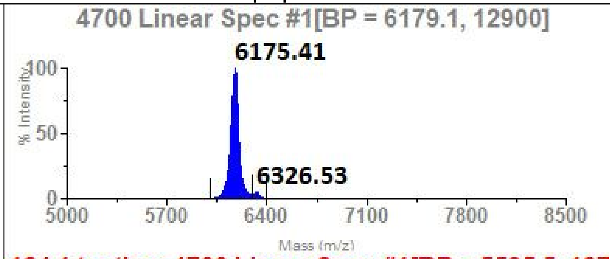
</td><td rowspan="2">Is processed by MrsT150-His</td></tr>
<tr><td>+MrsT150-His</td><td>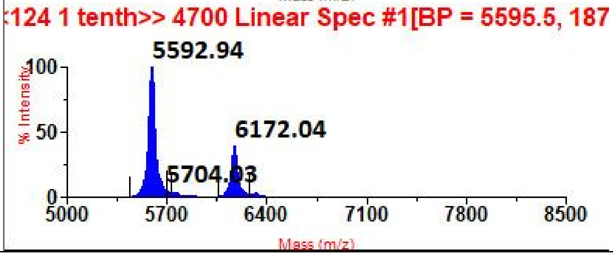
</td></tr>
</table>

S9. List of primers used in this study

	Sequence	Template	Result
fw	GACGGGTCTCCGGCGGCAGCATGTACTTTTACATTGCCTGGTG	pACYC His-MrsA + MrsM[1]	pACYC E-3A + MrsM
rv	GACGGGTCTCCCGCCATGTCTCCCGCACCTACTAACTTATCC		
fw	GACGGGTCTCCAGCGATGGAAGCAGCATGTACTTTTACATTG		pACYC D-5A + MrsM
rv	GACGGGTCTCCCGCTCCCGCACCTACTAACTTATCCATTTG		
fw	GACGGGTCTCCGCAGGCAGCATGTACTTTTACATTGCCTGGTG		pACYC E-3Q + MrsM
rv	GACGGGTCTCCCTGCATGTCTCCCGCACCTACTAACTTATCC		
fw	GACGGGTCTCCAAACATGGAAGCAGCATGTACTTTTACATTG		pACYC D-5N + MrsM
rv	GACGGGTCTCCGTTTCCCGCACCTACTAACTTATCCATTTG		
fw	GACGGGTCTCCATGGCGGCAGCATGTACTTTTACATTGCCTGGTG		pACYC E-3A D-5A + MrsM
rv	GACGGGTCTCCCCATCGCTCCCGCACCTACTAACTTATCCATTTGTG		
fw	GACGGGTCTCCATGCAGGCAGCATGTACTTTTACATTGCCTGGTG		pACYC E-3Q N-5A + MrsM
rv	GACGGGTCTCCGCATGTTTCCCGCACCTACTAACTTATCCATTTGTG		
fw	GACGGGTCTCCGAAAGCAGCATGTACTTTTACATTGCCTGGTGG		pACYC E-3K + MrsM
rv	GACGGGTCTCCTTTCATGTCTCCCGCACCTACTAACTTATCC		
fw	GACGGGTCTCCAAAGATGGAAGCAGCATGTACTTTTACATTGCCTG		pACYC D-5K + MrsM
rv	GACGGGTCTCCCTTTCCCGCACCTACTAACTTATCCATTTGTGC		
fw	GACGGGTCTCCAGACATGGAAGCATGTACTTTTACATTGCCTGGTGGCGG		pACYC AGDMEA + MrsM
rv	GACGGGTCTCCGTCTCCCGCTGCACCTACTAACTTATCCATTTGTGCTTCTTTCAGCTC		
fw	GACGGGTCTCCAAGCAGCAGCGTGTACTTTTACATTGCCTGGTGGCGG		pACYC DMEAAA + MrsM
rv	GACGGGTCTCCGCTTCCATGTCCGCACCTACTAACTTATCCATTTGTGCTTCTTTC		
fw	GACGGGTCTCGGAGCATGTACTTTTACATTGCCTGGG		pACYC ΔDMEA + MrsM
rv	GACGGGTCTCGGCTCCCGCACCTACTAACTTATCC		
fw	GACGGGTCTCGGAGCATGTACTTTTACATTGCCTGG		pACYC ΔDMEAA + MrsM
rv	GACGGGTCTCGGATGTACTTTTACATTGCCTGGTGG		
fw	GACGGGTCTCGGAGCATGTACTTTTACATTGCCTGG		pACYC ΔGDMEAA + MrsM
rv	GACGGGTCTCGCATCCCGCACCTACTAACTTATCC		
fw	GACGGGTCTCCCATGTACTTTTACATTGCCTGGTGGCGG		pACYC ΔA-1 + MrsM
rv	GACGGGTCTCCCATGCTTCCATGTCTCCCGCACCTAC		
fw	GACGGGTCTCCCGGACATGGAAGCAGCATGTACTTTTACATTGCC		pACYC ΔG-6 + MrsM
rv	GACGGGTCTCCTCCGCACCTACTAACTTATCCATTTGTGCTTCTTTC		
fw	GACGGGTCTCCTAGGTGCGTGTACTTTTACATTGCCTGGTGGCG		pACYC ΔK-11 + MrsM
rv	GACGGGTCTCCCCTACTAAATCCATTTGTGCTTCTTTCAGCTCACTGAATG		
fw	GACGGGTCTCCAAGCACAAATGGATAAGTTAGTAGGTGCGGGAG		pACYC ΔE-16 + MrsM
rv	GACGGGTCTCCGCTTTCAGCTCACTGAATGGGTTACCAGCTG		
fw	GACGGGTCTCCCAAGTGAGCTGAAAGAAGCACAAATGGATAAGTTAGTAG		pACYC ΔF-21 + MrsM
rv	GACGGGTCTCCCTTGGGTTACCAGCTGGATTTTGTGTAGAATTTTC		
fw	GACGGGTCTCCGCGTTAGTAGGTGCGGGAGACATGGAAG		pACYC K-11A + MrsM
rv	GACGGGTCTCCACGCATCCATTTGTGCTTCTTTCAGCTCACTGAA		
fw	GACGGGTCTCCAGCGGCACAAATGGATAAGTTAGTAGGTGCGGGAGAC		pACYC E-16A + MrsM
rv	GACGGGTCTCCCGCTTTCAGCTCACTGAATGGGTTACCAGCTG		
fw	GACGGGTCTCCAGCGAGTGAGCTGAAAGAAGCACAAATGGATAAGTTAG		pACYC F-21A + MrsM
rv	GACGGGTCTCCCGCTGGGTTACCAGCTGGATTTTGTGTAGAATTTTC		

References

(1) Viel, J. H.; Jaarsma, A. H.; Kuipers, O. P. Heterologous Expression of Mersacidin in Escherichia Coli Elucidates the Mode of Leader Processing. *ACS Synth. Biol.* **2021**, *10* (3), 600–608. https://doi.org/10.1021/acssynbio.0c00601.
